# BusR senses bipartite DNA binding motifs by a unique molecular ruler architecture

**DOI:** 10.1093/nar/gkab736

**Published:** 2021-08-25

**Authors:** Adrian M Bandera, Joseph Bartho, Katja Lammens, David Jan Drexler, Jasmin Kleinschwärzer, Karl-Peter Hopfner, Gregor Witte

**Affiliations:** Gene Center, Ludwig-Maximilians-Universität München, Feodor-Lynen-Str. 25, D-81377 München, Germany; Department of Biochemistry, Ludwig-Maximilians-Universität München, Feodor-Lynen-Str. 25, D-81377 München, Germany; Gene Center, Ludwig-Maximilians-Universität München, Feodor-Lynen-Str. 25, D-81377 München, Germany; Department of Biochemistry, Ludwig-Maximilians-Universität München, Feodor-Lynen-Str. 25, D-81377 München, Germany; Gene Center, Ludwig-Maximilians-Universität München, Feodor-Lynen-Str. 25, D-81377 München, Germany; Department of Biochemistry, Ludwig-Maximilians-Universität München, Feodor-Lynen-Str. 25, D-81377 München, Germany; Gene Center, Ludwig-Maximilians-Universität München, Feodor-Lynen-Str. 25, D-81377 München, Germany; Department of Biochemistry, Ludwig-Maximilians-Universität München, Feodor-Lynen-Str. 25, D-81377 München, Germany; Gene Center, Ludwig-Maximilians-Universität München, Feodor-Lynen-Str. 25, D-81377 München, Germany; Department of Biochemistry, Ludwig-Maximilians-Universität München, Feodor-Lynen-Str. 25, D-81377 München, Germany; Gene Center, Ludwig-Maximilians-Universität München, Feodor-Lynen-Str. 25, D-81377 München, Germany; Department of Biochemistry, Ludwig-Maximilians-Universität München, Feodor-Lynen-Str. 25, D-81377 München, Germany; Gene Center, Ludwig-Maximilians-Universität München, Feodor-Lynen-Str. 25, D-81377 München, Germany; Department of Biochemistry, Ludwig-Maximilians-Universität München, Feodor-Lynen-Str. 25, D-81377 München, Germany

## Abstract

The cyclic dinucleotide second messenger c-di-AMP is a major player in regulation of potassium homeostasis and osmolyte transport in a variety of bacteria. Along with various direct interactions with proteins such as potassium channels, the second messenger also specifically binds to transcription factors, thereby altering the processes in the cell on the transcriptional level. We here describe the structural and biochemical characterization of BusR from the human pathogen *Streptococcus agalactiae*. BusR is a member of a yet structurally uncharacterized subfamily of the GntR family of transcription factors that downregulates transcription of the genes for the BusA (OpuA) glycine-betaine transporter upon c-di-AMP binding. We report crystal structures of full-length BusR, its apo and c-di-AMP bound effector domain, as well as cryo-EM structures of BusR bound to its operator DNA. Our structural data, supported by biochemical and biophysical data, reveal that BusR utilizes a unique domain assembly with a tetrameric coiled-coil in between the binding platforms, serving as a molecular ruler to specifically recognize a 22 bp separated bipartite binding motif. Binding of c-di-AMP to BusR induces a shift in equilibrium from an inactivated towards an activated state that allows BusR to bind the target DNA, leading to transcriptional repression.

## Graphical Abstract

**Graphical Abstract:**
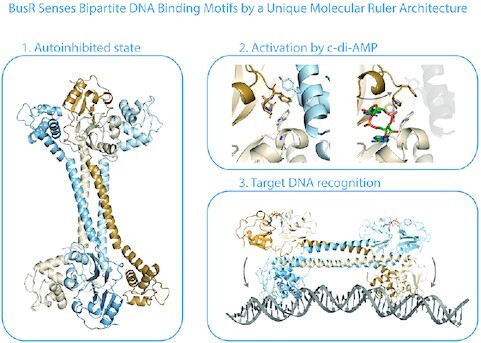
c-di-AMP mediated transcriptional control by BusR.

## INTRODUCTION

The bacterial cyclic dinucleotide second messenger c-di-AMP was discovered in 2008 in the crystal structure of the DNA-integrity scanning protein DisA (1). Initially, DisA and c-di-AMP were identified as a checkpoint protein or signal for maintaining DNA-integrity in *Bacillus subtilis*, and to be responsible for delay in sporulation upon DNA-damage (2,3). The identification of c-di-AMP initiated a new field in microbial signaling research, which has since become well-established. The role of c-di-AMP in the context of the bacterial cell is now increasingly clear, but many signaling pathways are still to be explored in detail (4). Aside from its role in DNA maintenance, c-di-AMP has been linked to cell wall integrity, potassium and osmotic homeostasis, virulence, sporulation and biofilm formation (5–7). The c-di-AMP pathway is present in many gram-positive bacteria and also archaea, and occurs in a plethora of pathogens including *Staphylococcus aureus* (including MRSA), *Bacillus anthracis*, *Listeria monocytogenes*, *Streptococcus pneumoniae* and *Mycobacterium tuberculosis*, among others (8–11).

In brief, the cyclic dinucleotide is synthesized by diadenylate cyclase domain (DAC) carrying proteins, can be bound by a variety of c-di-AMP receptors (proteins and riboswitches), and is either degraded by specific phosphodiesterases or is exported out of the cell. The group of DAC-proteins can be split up in three major classes, DacA, cdaS and DisA (1,12,13). Degradation of c-di-AMP is carried out by three families of phosphodiesterases (PDEs), namely PgpH-type PDEs belonging to the family of 7TMR membrane proteins with a cytosolic HD-domain that is the catalytically active PDE domain, the recently discovered actinobacterial manganese dependent PDE AtaC, and two types of DHH-type PDEs (14–17).

The correct concentration of c-di-AMP in cells is essential under normal growth conditions - both too low and too high concentrations result in severe effects on the viability of the cells (8,18–20). Altering c-di-AMP levels in the cells and thus disturbing its regulated processes has been shown to also alter resistance or susceptibility against antibiotics (21,22). For example, a methicillin resistant *S. aureus* strain that was depleted of c-di-AMP became sensitive again against β-lactam antibiotics (23). This renders the c-di-AMP pathway a remarkable target for antimicrobial therapies.

Once synthesized and available in the cells, c-di-AMP regulates many processes that are connected to K^+^-homeostasis and osmotic regulators such as compatible solute importers (24,25). A viable cell needs to balance its turgor to cope with the continuously changing osmolarity of its environment. The turgor needs to be efficiently controlled as upshifts or downshifts of osmolarity would rapidly lead to dehydration or cell lysis. One approach used by many organisms in response to hyperosmotic stress is to rapidly increase the uptake of ions (26). In this respect, c-di-AMP has been shown to control potassium uptake on multiple levels. c-di-AMP binds to different subunits of potassium transporters (e.g. KtrAB, KtrCD, KimA, CpaA, KhtTU) and decreases or increases uptake or export of ions, respectively (27–32). Furthermore, gene expression of potassium transporters has been shown to be under the control of c-di-AMP, either via a c-di-AMP sensitive riboswitch (e.g. kimA, ktrAB, kdpFABC) or via a two-component system (e.g. kdpABC via kdpDE) (25,33–35). In many organsims, uptake of potassium is the first response to osmotic upshift. Meanwhile, compatible solutes, e.g. proline or glycine-betaine, are either imported or synthesized. C-di-AMP deficient strains of *S. aureus*, *L. monocytogenes* or *S. agalactiae* have been shown to acquire mutations that affect the uptake of osmolytes (21,36,37). Furthermore, c-di-AMP has been shown to repress gene transcription for osmolyte uptake systems, e.g. *busA* from *S. agalactiae* and *Lactococcus lactis*, as well as to decrease the activity of osmolyte transporters like OpuC and *L. lactis* BusA (37–40). These findings show that c-di-AMP is a key regulator of osmolyte homeostasis that regulates osmolarity at multiple levels.

To get more detailed molecular insights into how these processes are regulated by c-di-AMP, we here focus on the role of c-di-AMP in transcriptional regulation. Aside from the TetR-like transcription factor DarR, BusR is the second transcription factor that has recently been linked to c-di-AMP (37,39,41). BusR is a member of the GntR family, which can be split into several subfamilies according to their C-terminal effector and oligomerization domains. Prominent subfamilies include FadR, HutC, MocR, YtrA and AraR (42–44). Based on the current classifications for these subfamilies BusR cannot be sorted into any of these and most likely comprises a subfamily of its own. BusR satisfies the overall domain architecture of the GntR family with its N-terminal winged helix-turn-helix motif (wHTH) and a C-terminal effector binding motif, an RCK_C domain (regulator of K^+^ conductance). This domain is known for binding c-di-AMP and is often utilized by c-di-AMP regulated potassium transporters (e.g. KtrA, KtrC, CpaA, KhtT) (27,28,32,45). In BusR, the wHTH and RCK_C domains are predicted to be connected by a coiled-coil region. *In vitro*, BusR represses the transcription of the *busA* operon in *L. lactis* in an osmolarity dependent manner (46,47). BusA is a glycine-betaine transporter crucial for responding to osmotic stress. A high amount of c-di-AMP leads to reduced expression of the transporter and thus less osmolyte uptake, while in the absence of c-di-AMP *busA* is constitutively expressed (37). Even though BusR is regulated by a second messenger which could imply a more global role, further targets of BusR have not yet been experimentally characterized. However, additional regulons for BusR in *L. lactis* subsp*. lactis* IO-1 were predicted by phylogenetic footprinting. These include *arsC*, an arsenic reductase, *hom (thrA)* and *thrB*. All three genes can be linked to osmotic homeostasis (48).

Here, we provide a biochemical and structural analysis of *Streptococcus agalactiae* BusR (SgaBusR). We present structures of apo, ligand bound and operator DNA bound states. We propose a mechanism for regulation and high binding specificity based on a molecular ruler guided site recognition, unseen in other GntR family members so far.

## MATERIALS AND METHODS

### Cloning, expression and purification of SgaBusR

The gene encoding BusR from *S. agalactiae* (DSM 16828) was cloned into a modified pET47b (Novagen) expression vector including an N-terminal (6x)His-MBP tag and a PreScission protease cleavage site (pET47b-MBP) (49). Point mutations were introduced by PCR amplification. For protein expression transformed *Escherichia coli* (DE3) Rosetta were grown in Turbo Broth™ (Molecular Dimensions) at 37°C to OD_600_ of 0.8, cooled down to 18°C and induced with 0.2 mM Isopropyl-β-d-thiogalactopyranosid (IPTG) at OD_600_ of 1.3 for overnight expression.

For selenomethionine expression, methionine auxotroph B834 Rosetta cells were grown in SelenoMethionine Medium Complete (Molecular Dimensions). Protein expression was induced at OD_600_ of 0.8. Purification of selenomethionine substituted protein was performed according to the purification of native protein with the exception of 5 mM β-mercaptoethanol in all buffers.

Cell pellets with overexpressed His-MBP-BusR constructs were resuspended in buffer A (100 mM NaP_i_ pH 6.5, 200 mM NaCl, 20 mM imidazole, 5% glycerol) and lysed by sonication. The lysate was cleared by centrifugation and filtration and loaded onto a 5 ml Ni-NTA column (Cytiva). The sample was washed with buffer HS (100 mM NaP_i_ pH 6.5, 1 M NaCl, 20 mM imidazole, 5% (v/v) glycerol) and buffer B_50_ (100 mM NaP_i_ pH 6.5, 200 mM NaCl, 50 mM imidazole, 5% (v/v) glycerol) and eluted with buffer B (100 mM NaP_i_ pH 6.5, 200 mM NaCl, 500 mM imidazole, 5% (v/v) glycerol). PreScission protease was added to the eluent prior to dialysis against buffer A at 8°C overnight. The protein was loaded onto a 5 ml Heparin column (Cytiva) and eluted using buffer HS and directly passed through a 5 ml Ni-NTA column (Cytiva) for removal of any non-digested species. The flow-through was collected and as a polishing step size exclusion chromatography (SEC) was conducted using a HiLoad Superdex S200 column (Cytiva, S75 for small constructs) preequilibrated in buffer SEC (30 mM NaP_i_ pH 6.5, 200 mM NaCl, 3% (v/v) glycerol). For all samples intended for crystallization trials buffer H (30 mM HEPES pH 7.0, 200 mM NaCl) was used for SEC instead. All steps were analyzed by SDS-PAGE. Protein concentration was determined spectrophotometrically using the theoretical molar extinction coefficient calculated from amino acid composition (50). All concentrations are given in tetramers unless otherwise stated. The protein was concentrated to 10 mg/ml and flash frozen in liquid nitrogen and stored at -80°C. RCK_C constructs were purified in an altered way. After Ni-NTA, samples were directly applied to size exclusion chromatography using buffer H. For crystallization of the ligand bound state, the His-tag was removed by PreScission protease (Cytiva) cleavage prior to size exclusion chromatography.

### Small-angle X-ray scattering

Small-angle scattering data were either obtained by batch mode data collection, i.e. a sample (between 1 and 14 mg/ml) and the respective buffer (30 mM HEPES pH 7.5, 100 mM NaCl) were measured in alternating steps, or in size-exclusion chromatography coupled SAXS (SEC-SAXS) with the running buffer as reference. Full length BusR was measured in 20 mM HEPES pH 7.5, 200 mM NaCl, 3% glycerol (v/v). BusR:c-di-AMP:pAB1 was measured in 20 mM HEPES pH 6.5, 100 mM NaCl, 3% glycerol (v/v). Measurements were done at the P12 SAXS beamline at the PETRA III storage ring (EMBL Hamburg, DESY Hamburg). Data were processed using CHROMIXS and PRIMUS of the ATSAS suite (51) and analyzed as reviewed in (52,53). Data were checked for aggregation by Guinier-plot analysis (Guinier approximation in the s*R_G_ < 1.3 region). Theoretical scattering curves were calculated from atomic models using CRYSOL (51). SAXS data have been deposited into the SASBDB database with accession numbers SASDK74 (RCK_C domain dimer), SASDK84 (BusR) and SASDK94 (BusR:cdiAMP:pAB1-complex).

### Crystallization and structure determination

All crystals were obtained using hanging-drop vapor diffusion in a 1:1 mixture of purification buffer and crystallization solution at 20°C. Full-length selenomethionine substituted BusR crystals were grown at 7 mg/ml in 150 mM magnesium-acetate and 6% D + trehalose. Crystals were subjected to mother liquor containing 35% ethylene glycol and subsequently flash frozen in liquid nitrogen. Apo His_6_-RCK_C crystals were grown at 6 mg/mL in 0.1 M Bis–Tris pH 6.5, 18% (w/v) PEG MME 5000. For the ligand bound state 4.5 mg/mL BusR RCK_C (no His_6_-tag) was co-crystallized with 1 mM c-di-AMP in 100 mM sodium-acetate pH 4.6, 3% PEG 4000. 30% PEG 400 was used as cryoprotectant for both crystals. Crystals were measured at the EMBL Hamburg P13 and P14 beamlines at PETRA III storage ring (DESY Hamburg) at 100 K. Data were indexed, integrated and scaled using XDS/XSCALE (54). HKL2MAP (55)/SHELX (56) were used for experimental phasing of the full length dataset collected at selenium peak wavelength. Initial model building was done automatically using Buccaneer (57). Manual building was continued in COOT (58) and refinement was performed in PHENIX (59). Phases for the RCK_C constructs were obtained by molecular replacement using the RCK_C domain of the full-length structure as search model in PHASER/PHENIX (59). Data collection and refinement statistics are provided in Supplementary Table S1. Buried surface area between substructures was calculated using the PISA server (60).

### Cryo-electron microscopy

A 152 bp long DNA substrate containing pAB1 and pAB2 (called pAB) was amplified from genomic DNA by PCR and purified by PCR clean up (Macherey & Nagel). 0.4 mg/ml of BusR were incubated with 0.2 mg/mL DNA and 100 μM c-di-AMP in 20 mM HEPES pH 6.5, 100 mM NaCl. Prior to grid preparation β-octyl glucoside was added to a final concentration of 0.05%. 4.5 μl of sample was applied to plasma cleaned (GloCube, Quorum) QuantiFoil R2/1 200 mesh holey carbon grids (Quantifoil) and plunge frozen in liquid ethane using a Leica EM GP. Data was acquired using a Titan Krios transmission electron microscope (Thermo Fisher Scientific) operated at 300 keV, with a Gatan K2 Summit detector operated in counting mode, and Gatan GIF Quantum energy filter. EPU software (TFS) was used for automated acquisition. 9381 micrographs with a nominal magnification of 130 000×, calibrated pixel size of 1.046 Å, defocus range of −1.1 to −2.6 μm, and a total dose of 45 e^–^/Å^2^ over 40 frames were collected. For data processing all micrographs were aligned using Motioncor2 (61). The subsequent steps were done in cryoSPARC v3.2 (62). Local CTF estimation was done with CTFFIND4 implemented in cryoSPARC. Initial particles were picked using blob picker. These particles were 2D classified, and high quality and diverse classes were selected and low pass filtered to 20 Å for training of the topaz neural network picker embedded in cryoSPARC (63,64). The topaz picked particles were extracted and resubjected to 2D classification and topaz training. Final extraction was done with a box size of 256 pixels and particles were subjected to 2D classification. Classes of good resolution were selected and used to generate an initial 3D model (C1). The best volume was used as a reference in Relion 3.1 for further 3D classification (65). The best 3D classes were selected and used for 3D refinement. After polishing and CTF Refinement, fixed angle 3D classification was performed using a mask that excludes the protruding DNA. 3D refinement and post processing using the same mask resulted in a final resolution estimate of 4.46 Å. Model building was done by using the crystallographic full-length structure of BusR and the c-di-AMP bound RCK_C domain structure (both this work). These models were rigid body fitted into the cryo-EM map using UCSF Chimera. Coot was used for further building. The linker between the coiled-coils and the RCK_C domain was manually built into the density. An ideal B-DNA based on the sequence of pAB2 was generated and bent to fit the density. Data collection and refinement statistics are provided in Supplementary Table S2.

### Static-light scattering

Size exclusion chromatography coupled right angle light scattering (SEC-RALS) was performed using an ÄKTA micro, a Superdex S200 10/300 increase column (Cytiva), a right-angle light scattering detector and a refractive index detector (Malvern/Viscotek). Assays were performed at 20°C in buffer K (30 mM KP_i_ pH 6.5, 20 mM KCl) for BusR-DNA or DNA alone and buffer SEC for BusR alone or with c-di-AMP. BSA was used as a standard for calibration. Data evaluation was done with the OmniSEC software package (Malvern).

### Electrophoretic mobility shift assays

Electrophoretic mobility shift assay (EMSA) was done with 5′-6-FAM labelled DNA (Supplementary Table S3). 6% polyacrylamide gels were cast in 100 mM KP_i_ pH 6.5 and pre-run at 80 V for 90 min at 8°C. For detailed concentrations refer to the figure legends. BusR, DNA and nucleotides were incubated in 100 mM KP_i_ pH 6.5, 20 mM KCl for 30 min at 20°C before being separated on gel for 90 min at 8°C and 45 V. Gels were imaged using a Typhoon imager (Cytiva). For EMSAs with pAB containing both binding sites the respective promotor region of *busA* was amplified by PCR from genomic DNA and cloned into a pUC19 vector. For the control the pAB1 GAC binding sites were deleted by site directed mutagenesis. The DNA was then amplified with 5′-6-FAM labelled primers for subsequent use in EMSAs.

### Isothermal titration calorimetry

Isothermal titration calorimetry experiments were conducted in buffer SEC using protein concentrations of 20 μM and a 20-fold excess of ligand in the syringe. The experiment was performed at 20°C, consisting of 19 injections of 2 μl, spaced 150 s apart. Control experiments were done by titrating buffer to buffer and ligand to buffer and were subtracted from the measurement using the Malvern software package. The binding parameters represent average values with SD (*n* = 3).

### Surface plasmon resonance

For surface plasmon resonance (SPR) experiments (Biacore X100, Cytiva) neutravidin was coupled to flow cell 1 and 2 of a CM5 chip using amino reactive EDC/NHS coupling chemistry in HBS-EP + buffer (150 mM NaCl, 10 mM HEPES–NaOH pH 7.4, 3 mM EDTA, 0.05% v/v surfactant P20). Biotin labelled pAB1 sequence was coupled to flow cell 2 and an unspecific DNA of equal length was coupled to flow cell 1 (serving as a reference). Raw data shown are reference subtracted (FC2-FC1). Assays were conducted in buffer S (100 mM NaP_i_ pH 6.5, 20 mM KCl, 200 mM NaCl, 3 mM EDTA, 0.05% v/v surfactant P20) and steady-state affinities were derived from single cycle kinetic experiments using the Biacore X100 evaluation software.

## RESULTS AND DISCUSSION

### Oligomerization and c-di-AMP binding

**Figure 1.**
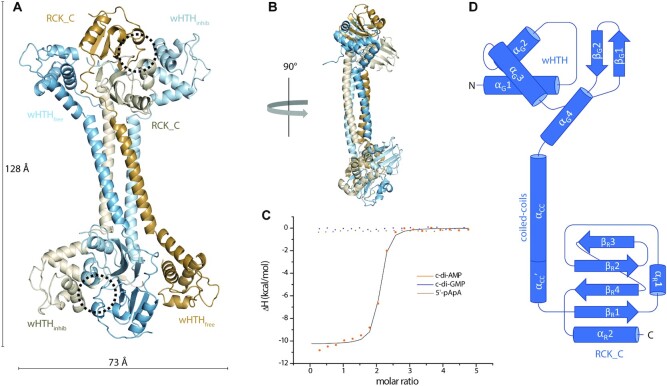
Crystal structure of apo *S. agalactiae* BusR and ligand binding. (**A**) Cartoon representation of the tetrameric crystal structure of apo BusR, colored by chain. The respective domains are labelled to illustrate the antiparallel head-to-tail arrangement. The ligand binding pocket is highlighted by a dashed circle. (**B**) Side view of the apo crystal structure, rotated by 90°. (**C**) Binding curve and fits of ITC measurements of BusR titrated with c-di-AMP (orange, *K*_D_= 112 ± 7 nM), 5′-pApA (brown, no binding) and c-di-GMP (blue, no binding) (*n* = 3). (**D**) Schematic overview of the secondary structure of a single BusR monomer from the apo crystal structure. The helix elongation of the central coiled-coils labelled α_CC_′ is only present in the light blue and light brown colored monomers (see panel A and B), while unstructured in the others.

BusR from *S. agalactiae* has been described to negatively regulate expression of the osmolyte uptake system BusA in a c-di-AMP dependent manner. Our aim is to understand the molecular mechanisms how BusR translates c-di-AMP levels to reduced expression of BusA. C-di-AMP binding capabilities of BusR including specificities have been demonstrated by DRaCALA experiments before, but neither binding affinities nor stoichiometry or its mechanism of regulation are yet known (39). To this end, we expressed and purified *S. agalactiae* BusR to homogeneity. BusR elutes as a single monodisperse peak from a size-exclusion chromatography column at an elution volume that is incompatible with a monomeric species. We therefore analyzed BusR without ligand (apo) and BusR with c-di-AMP (cplx) by SEC-coupled static light scattering and determined a molecular weight of BusR of Mw(apo) = 97.0 kDa (Mw^theoretical^ = 23.8 kDa) and Mw(cplx) = 96.3 kDa in the c-di-AMP bound state (Supplementary Figure S1). Thus, BusR is a stable tetramer in solution, in agreement with SAXS data (Supplementary Figure S2), and the oligomeric state is not altered upon binding of the ligand c-di-AMP. To thermodynamically characterize binding of c-di-AMP to BusR we performed isothermal titration calorimetry (ITC) and titrated BusR with the putative ligand c-di-AMP, and c-di-GMP and 5′-pApA as controls. The resulting ITC data convincingly show specific and high affinity binding of c-di-AMP even in high phosphate and high salt buffer (that is needed as c-di-AMP decreases BusR’s stability in absence of DNA), with a dissociation constant *K*_D_ = 112 ± 7 nM and a stoichiometry of *n* = 1.7. This indicates that one tetramer of BusR binds two molecules of c-di-AMP (Figure 1C, Supplementary Figure S3).

### Crystal structure of BusR

In order to understand the molecular mechanisms of BusR signaling we crystallized selenomethionine substituted full-length BusR and solved its structure to 2.8 Å resolution by single-wavelength anomalous diffraction experiment. As the protein was recombinantly expressed and purified from *E. coli*, that lack DAC domain proteins and the entire c-di-AMP pathway, our structure represents the apo state. The asymmetric unit contains the physiological functional tetramer of BusR (Figure 1A). Each monomer contains an N-terminal wHTH domain and a C-terminal effector binding RCK_C domain. Both domains are linked by a long α-helix (α_CC_). The DNA binding domain consists of a two stranded winged helix-turn-helix motif, following the pattern of α_G_1–α_G_2–α_G_3–β_G_1–β_G_2, followed by an additional C-terminal helix α_G_4. The four central α_CC_-helices of the four monomers constitute a four-stranded coiled-coil motif, thereby forming a robust tetramerization interface. The four monomers are arranged in a 2-parallel 2-antiparallel head-to-tail arrangement, forming an overall shape resembling a dumbbell. The RCK_C domains form a dimer on each side of the BusR dumbbell. Each of these dimers is flanked by the two wHTH motifs of the antiparallel strands. The interfaces between the RCK_C dimer and its neighbouring wHTH motifs differ significantly resulting in two distinct DNA binding domain arrangements that we refer to as wHTH_inhib_ and wHTH_free_. The interface between the RCK_C domain and wHTH_inhib_ is approx. 50% larger in buried surface area (wHTH_free_: area = 902 Å^2^ versus wHTH_inhib_: area = 1353 Å^2^). The RCK_C – wHTH_inhib_ interface is stabilized by a hydrophobic region around Trp159. In contrast, wHTH_free_ is only loosely connected to the neighbouring RCK_C via a protruding loop connecting α_CC_ and the distal RCK_C. The significance of this observation will be addressed below.

The overall shape of BusR with two separated DNA-binding platforms, led us to the idea that the interconnecting coiled-coil domain might serve as a molecular ruler to increase binding site specificity, and the observation of two different wHTH versus RCK_C orientations on each side of the dumbbell suggests that these interactions might be taking part in c-di-AMP mediated activation of BusR.

### Binding of c-di-AMP to BusR increases affinity for operator DNA

We next addressed the question of how c-di-AMP alters the DNA binding properties of BusR. Using DNA footprinting experiments is has been recently shown that there are two quite large binding sequences of BusR in the promotor sequence of *busA* (37). One binding site is closer to the start codon (here named pAB1) and the other binding site overlaps with the −35 and −10 element (pAB2). A comparison of these two sequences shows that they share a motif with 22 nucleotides of random sequence flanked by an inverted repeat: 5′-GAC-N_22_-GTC-3′. We observed no major differences in the binding of these sequences in our binding experiments with BusR (Supplementary Figure S4B) and therefore confined ourselves to pAB1. This does not imply that the binding sites may not have different roles *in vivo*.

**Figure 2.**
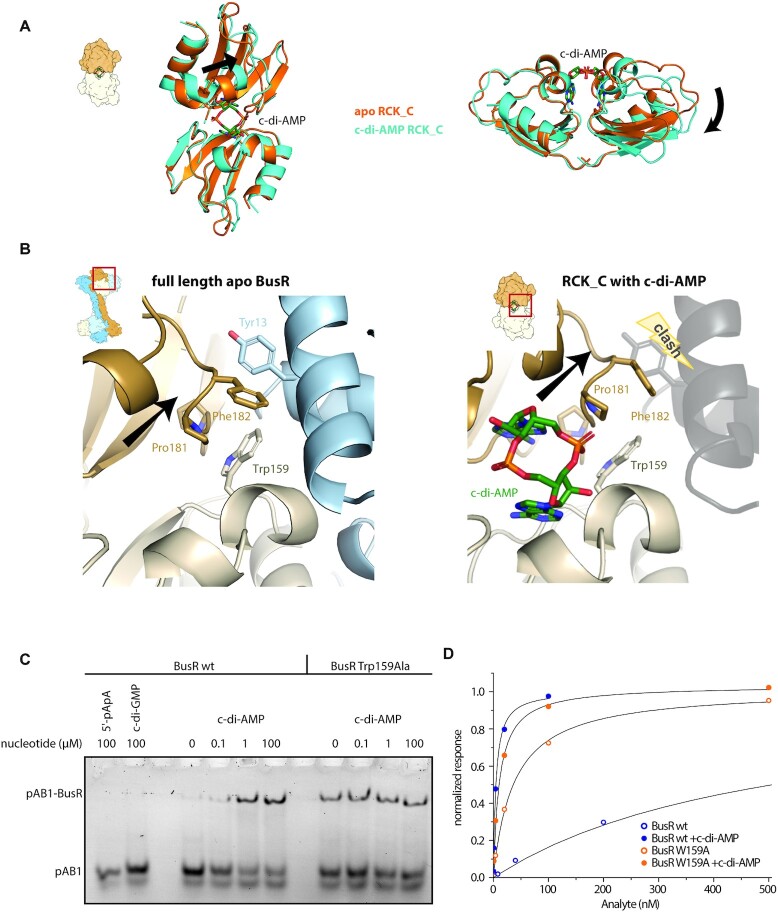
Release of autoinhibition upon c-di-AMP binding to BusR. (**A**) Front and side view of superimposed crystal structures of apo RCK_C domain (orange) and ligand bound RCK_C domain (cyan, c-di-AMP in dark green). Ligand induced movement of the monomers in respect to each other is indicated by a black arrow (1.31 Å rmsd for apo RCK_C compared to ligand bound RCK_C). (**B**) Close-up on RCK_C – wHTH_inhib_ interface in the full-length (left) and c-di-AMP bound RCK_C (right) crystal structure. In the full-length apo state a tight interface is formed around residues Trp159 (RCK_C chain A, light brown), Pro179-Phe182 (RCK_C chain B, dark brown) and Tyr13 (wHTH_inhib_, blue). Binding of c-di-AMP induces rotational movement (black arrow) of the RCK_C monomers in respect to each other that would cause sterical clashes with helices α_G_1 and α_G_3 (grey and transparent), which frees the wHTH_inhib_ domain for subsequent DNA binding. (**C**) EMSA experiments: c-di-AMP titrated to 100 nM wt BusR leads to increased affinity for operator DNA (20 nM), while related nucleotides show no effect. The mutation of Trp159Ala disturbs the RCK_C–wHTH_inhib_ interface and thereby allows for DNA binding of BusR in absence of c-di-AMP (*n* = 3). (**D**) Steady-state affinity binding fits of SPR measurements using a single site binding model. Titration of BusR and BusR Trp159Ala to pAB1 in presence and absence of c-di-AMP (10 μM) (*n* = 3).

So far, it has been shown that expression levels of BusA are negatively regulated by BusR and c-di-AMP *in vivo* (37). To further characterize BusR biding *in vitro* we performed electrophoretic mobility shift assays (EMSA). Upon increasing concentration of c-di-AMP in presence of BusR and pAB1, the free DNA is shifted towards a complex band, showing that the affinity of BusR for pAB1 is strongly increased in presence of the second messenger (Figure 2C). At these conditions maximum binding is achieved at 1 μM of c-di-AMP. This capability to sense low c-di-AMP concentrations allows for a sensitive and rapid regulation of BusR activity (i.e. repression of *busA*). SPR experiments confirm this result and display an increase in binding affinity of BusR for pAB1 from *K*_D_ = 577 ± 55 nM in absence to *K*_D_ = 5 ± 0.4 nM in presence of c-di-AMP (Figure 2D, Supplementary Figure S5). We assume that the dissociation constant for this binding likely represents an upper limit due to the influence of the high salt and phosphate condition used in the experimental setup. The effect of c-di-AMP is highly specific, as control experiments with c-di-GMP and 5′-pApA show no impact on the affinity of BusR to DNA in an EMSA (Figure 2C), in line with the results from ITC. This finding raised the question of how c-di-AMP binding alters the affinity of BusR for DNA on a molecular basis and whether the observed RCK_C – wHTH_inhib_ interface is a key element in this regulatory event.

### Atomic resolution structure of the RCK_C domain

While our full-length structure revealed the overall domain architecture of BusR, it lacks the necessary resolution to thoroughly describe ligand binding. In order to understand the effect of c-di-AMP on DNA binding of BusR we focused on the isolated RCK_C domains. We purified and crystallized a construct comprising only the RCK_C domain (Leu135 to end) and solved its ligand-free and c-di-AMP bound structure to 1 and 1.2 Å resolution, respectively. The crystal of the apo state only contains one monomer in the asymmetric unit. However, a symmetry related copy is present that generates the dimeric assembly as common to RCK_C domains and as observed in our full-length structure. To probe whether the BusR RCK_C domain is monomeric in solution and dimerizes upon c-di-AMP binding we conducted small-angle X-ray scattering measurements. The scattering data show that the RCK_C domain on its own is dimeric in solution even in the absence of c-di-AMP (Supplementary Figure S2G). The crystal of the RCK_C domain in the c-di-AMP bound state contains the physiological dimer in the asymmetric unit with one moiety of c-di-AMP bound. The c-di-AMP binding pocket is localized in the cleft between α_R_1 of the two RCK_C subunits in a conserved hydrophobic and narrow pocket formed by residues Ile153, Gly154, Val158, Ala164, Thr165, Ile166, Pro181 and the main chain of Trp159 and Gly180. Residues Asn157 and His160 form a polar rim around the pocket (Supplementary Figure S6). The high-resolution structure of the RCK_C domain explains very well the high specificity of BusR for c-di-AMP and how it discriminates its ligand even from structurally closely related molecules such as its linearized variant 5′-pApA, or c-di-GMP. The adenine base, especially its amino group forms hydrogen bonds to the peptide backbone of Ile166 while Ala164 at the same time prevents a larger group at C2 of the adenine base, thus preventing c-di-GMP from binding. The 2′-OH group of the ribose is coordinated via a hydrogen bond by Asp157 and the phosphate group forms a hydrogen bond with His160. The individual monomers are identical and the resulting assembly is symmetric, therefore all protein-ligand interactions are mirrored on the opposite site of c-di-AMP (Supplementary Figure S6).

### Autoinhibition mediated by the RCK_C domain

The large interface between wHTH_inhib_ and the RCK_C domain observed in our full-length structure and the fact that it positions the ligand binding site in close proximity to one of the wHTH motifs, raises the idea that it might play a role in regulation. Furthermore, in the observed conformation, the DNA recognition helix α_G_3 of wHTH_inhib_ is buried in the interface and thus incapable to bind to DNA. At the core of the interface the residues Trp159 (RCK_C, chain A), Pro181, Phe182 (RCK_C, chain B) and Tyr13 (wHTH_inhib_) are forming a hydrophobic pocket (Supplementary Figure S7). Intriguingly, these residues of the hydrophobic patch are highly conserved among homologs of BusR (Supplementary Figure S8). This may not be surprising for Tyr13 that is involved in DNA binding or Pro181 that is an integral part of the c-di-AMP binding pocket. However, it is notable for residues Trp159 and Phe182 as their sidechains are not involved in either one. Phe182 is often found to be replaced by a tyrosine, which however is a residue of comparable chemical properties.

The apo and c-di-AMP bound crystal structure of the RCK_C domain show that c-di-AMP binding induces a subtle rotation of the RCK_C domains that results in a widening of the binding pocket (Figure 2A). This movement pushes residues Pro179, Gly180, Pro181 and Phe182 (chain A) further out in respect to Trp159 (chain B) of the opposing chain. When we transfer this movement to the full-length structure this has two implications. Firstly, it weakens the hydrophobic patch and secondly, it leads to steric clashes of Pro181 and Phe182 with wHTH_inhib_, especially Tyr13 (Figure 2B). Thus, it seems that this interface locks wHTH_inhib_ in an autoinhibited state in absence of c-di-AMP, restricting movement of wHTH_inhib_ and thus impairing high affinity binding to the operator sequence.

To test this hypothesis and the relevance of this interface *in vitro* we mutated Trp159 to alanine, thereby weakening the hydrophobic patch. Indeed, the Trp159Ala mutant shows a c-di-AMP uncoupled DNA-binding: The mutant has higher affinity for its target DNA in absence of c-di-AMP in EMSAs and SPR measurements compared to wildtype BusR, while addition of c-di-AMP to the mutant affects the DNA affinity only marginally (Figure 2C, D). In comparison, wt BusR requires presence of 1 μM c-di-AMP to reach the same shift in EMSA as Trp159Ala (no c-di-AMP).

The conservation of the hydrophobic pocket, the high-resolution crystal structures of the apo and ligand bound state, and the c-di-AMP decoupled elevated affinity of the Trp159Ala mutant indicate that the hydrophobic patch surrounding Trp159 is important for signaling. It arrests BusR in an autoinhibited state that is released upon binding of c-di-AMP to allow for DNA binding and subsequent transcriptional control. To our knowledge this mechanism is different from other RCK_C domains and unique to BusR, which further becomes apparent by the fact that Trp159 and Phe182 are not conserved in paralogous and structurally highly similar RCK_C domains as for example *S. aureus* KtrA (pdb code 4xtt, (66)) (Supplementary Figure S9).

### DNA binding

**Figure 3.**
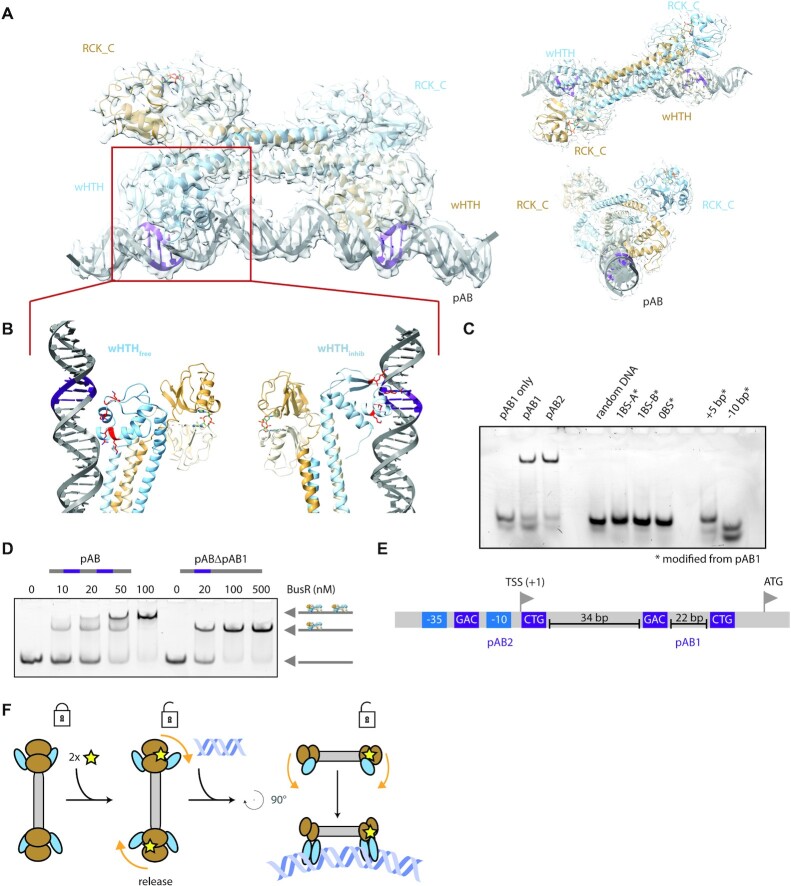
BusR bound to operator DNA. (**A**) cryoEM structure of BusR bound to operator DNA pAB depicted as side view (left), top view (top right) and front view (bottom right). The binding motifs are colored in purple. Large domain rearrangement occurs from apo to DNA bound BusR. The RCK_C–wHTH_inhib_ interface is broken and all four wHTH motifs rotate backwards to face and bind the DNA. (**B**) The DNA binding domain shows great variability in its interaction with the DNA. The wHTH_free_ rather interacts with the phosphate backbone instead of the bases (left part, for reasons of better visibility wHTH_inhib_ is not shown), whereas wHTH_inhib_ is deeply buried in the major groove with its wing bound to the minor groove (right part, wHTH_free_ is not shown). (**C**) BusR distinguishes its native substrate (pAB1 or pAB2) from non-target DNA. Alterations to the DNA severely disrupt binding of BusR in EMSAs. Deletion of one (BS-A or BS-B) or both binding motifs (0BS) from pAB1 abolishes DNA binding in EMSAs. Also changes to the position of the binding motifs, either by elongation of the DNA in between the binding motifs by 5 base pairs (+5 bp) or by shortening of the distance in between by 10 base pairs (−10 bp) prevents BusR from recognizing its substrate (BusR = 100 nM, DNA = 20 nM, c-di-AMP = 10 μM, *n* = 3). (**D**) EMSA with the promoter region containing both pAB1 and pAB2. Two complex bands are observed upon titration of BusR. A control with only one binding site present results in the lower complex band, indicating that this is a BusR:DNA complex of 1:1 stochiometry and linear instead of looped DNA (20 nM DNA, 10 μM c-di-AMP; *n* = 3). (**E**) Schematic overview of the two binding sites of BusR within the promotor region of BusA. Included are the −35 and −10 transcriptional elements, the transcriptional start site (TSS) and the start codon (ATG). (**F**) Schematic representation of the domain rearrangement and mechanism. Two molecules of c-di-AMP bind to the RCK_C domains. Consequently, the adjacent wHTH_inhib_ domain is released and can now freely rotate towards the DNA. Upon binding the DNA is bent by 18°.

The idea of a molecular ruler in combination with two sides of regulated (i.e. partially flexible) DNA-binding domains in a transcription factor render the structure of the dsDNA-bound BusR even more interesting. Thus, to visualize this ternary complex of c-di-AMP activated BusR on dsDNA, we solved two complex structures by single-particle cryo-electron microscopy. One structure contains BusR bound to pAB1 at a resolution of 7.1 Å (Supplementary Figure S10) and the other structure was determined using the whole promotor sequence (pAB, two BusR binding sites). In the reconstruction only BusR and the DNA in proximity to the binding site are visible and thus pAB1- and pAB2-bound BusR cannot be distinguished. However, the latter structure yields data up to 4.5 Å resolution and thus provides more details in the protein domains (Supplementary Figure S11). The higher resolution likely resulted from thinner ice after plunge freezing of the larger sample. As expected, BusR binds to the DNA as a tetramer, which reflects the oligomeric assembly we observed by SEC-RALS and SAXS in solution (Supplementary Figures S1 and S2). Compared to the crystal structure, BusR undergoes major structural rearrangements upon binding to dsDNA (Figure 3A). The central coiled-coils align in an ‘X’-like fashion relative to the dsDNA. BusR has a strikingly asymmetric surface charge distribution, with one predominantly positively charged side and a mainly negative charge on the opposite side (Supplementary Figure S12). This positively charged side of the BusR is facing the negatively charged DNA in the complex. In our structure, BusR induces a bending of the DNA by ∼18°. The DNA is also slightly bend to the side, thereby breaking the C2 symmetry of the complex.

C-di-AMP can be unambiguously identified in the ligand binding site within the RCK_C domain and the RCK_C–wHTH_inhib_ interface surrounding Trp159 is completely dissolved. In comparison to the crystal structure, the RCK_C domains tilt by 70° relative to the coiled-coils away from wHTH_inhib_ in the direction of wHTH_free_. This rotation of the RCK_C dimer creates space for wHTH_inhib_ to undergo a rotation of 60° towards the DNA. The rotation of wHTH_inhib_ allows the helices α_CC_ and α_G_4, which are interrupted in the apo state, to reseal to a single prolonged α-helix, spanning the entire molecule (video abstract). This brings the two equivalent DNA binding domains into the same position as the two binding motifs (5′-GAC-3′) that are spaced apart by three turns of DNA. While the resolution of the EM density is not good enough to determine the register of the DNA directly, it can be deduced from the lower resolution BusR:c-di-AMP:pAB1 structure, as the whole dsDNA density (46 bp) is visible. The Helices α_G_2 and α_G_3 of wHTH_inhib_ plunge deep into the major groove, in close contact to the motif sequence, while the ‘wing’ of wHTH_inhib_ (loop between β_G_1 and β_G_2) reaches into the minor groove (Figure 3B). We mutated residues that are close to the binding motif and are likely to interact with the DNA. These mutants, Lys36Ala and Arg38Ala (α_G_2), Arg53Ala and Lys54Ala (α_G_3) and Gly70Ile and Gly72Ile (loop between β_G_2 and β_G_3) all show a severely diminished binding in EMSAs (Supplementary Figure S3), while behaving identical to the wildtype during purification, proving their importance for binding site recognition. In the reverse experiment, mutating one (or both) of the two binding motifs to a random nucleotide sequence of equal length, BusR binding in EMSAs is also heavily compromised, indicating the importance of the 5′-GAC-3′ motif in the binding site (Figure 3C).

While the release of wHTH_inhib_ leads to a sequence specific binding, wHTH_free_ contributes to the protein-DNA interaction differently. The second DNA binding domain undergoes a major rearrangement: It rotates alongside the RCK_C domains by 90°. In this complex, wHTH_free_ faces the DNA in the same orientation as wHTH_inhib_ but is positioned one quarter of a DNA turn (90°) further inwards, towards the middle of the DNA sequence (Figure 3A, Supplementary Figure S4A). Thus, wHTH_free_ is not in direct proximity to the binding motif. The whole domain is slightly displaced and further away from the DNA and the wing does not reach into the minor groove (Figure 3B). Based on our structure, wHTH_free_ appears to be limited to phosphate backbone contacts. Our structure suggests that wHTH_free_ contributes in a rather sequence independent binding mode. This hypothesis is supported by sequence alignments of the four so far experimentally determined binding sequences of BusR (*S. agalactiae, L. lactis* and *Tetragenococcus halophilus)* showing a highly conserved target binding motif 5′-GAC-3′ for wHTH_inhib_, while the wHTH_free_ binding region is not very conserved (Supplementary Figure S4A), and also differs between pAB1 and pAB2.

As shown above, the two binding motif sequences are crucial for recognition by BusR, but even more striking is the need for their relative positioning in respect to each other. The distance between these two sites is 22 bp for all the four experimentally determined binding sequences. Including both flanking binding sequences, it sums up to three turns of the DNA double helix. This leads to positioning of both binding sequences facing to the same side of the DNA. It is tempting to speculate that the coiled-coil spacer serves as a molecular ruler to increase the specificity of BusR to specific sites in fixed distance to each other. To experimentally address the relevance of this spacing, we used dsDNA that contains additional 5 bp in the middle between the binding sequences in EMSAs. The +5 bp not only increase the distance between the binding motifs but turns the around on the dsDNA by 180° with respect to each other. Neither this DNA-segment is recognized by BusR, nor a −10 bp DNA with shorter middle segment, having both binding sequences facing to the same direction again but closer together (Figure 3C). This illustrates that both the recognition of the correct DNA sequence and the relative spacing of these sites is essential for BusR-DNA binding. In this sense, the coiled-coils in the center of BusR resemble a molecular ruler, adding a second layer of specificity to the transcription factor.

### BusR−DNA complex organization

The recently described tetrameric GntR family transcription factor Atu1419 from *Agrobacterium fabrum* binds to two binding sites that are 190 bp apart and bends the DNA around the protein causing DNA loop repression (67). This raises the idea that BusR might induce DNA loop formation, as well. To analyze BusR with respect to this looping option we performed cryo-EM with a rather long dsDNA containing both binding sites as substrate for cryoEM (BusR:c-di-AMP:pAB). In this dataset we could observe free DNA, and DNA with one or two copies of BusR. We could not identify any loop formation in the micrographs nor during further processing (e.g. no 2D classes with respective assemblies, S11B). In parallel, we tested the ability of BusR to bind to multiple copies of its operator dsDNA sequence by SEC-RALS. In presence of a 3-fold excess of pAB1 towards BusR we still observe only a 1:1 stoichiometry (Supplementary Figure S1D). Furthermore, we conducted band shift assays with dsDNA spanning both binding sites (Figure 3D). In presence of BusR two complex bands can be observed and increasing concentration of BusR leads to a shift of the lower complex band towards the upper complex band. While the upper complex band most likely represents a 2:1 BusR-DNA complex, the lower complex band can represent a linear or looped BusR-DNA complex. As a control we tested the same substrate lacking one binding site (ΔpAB1) by mutating both its GAC binding motifs, thus preventing loop formation. Band shifts with ΔpAB1 result in the loss of the upper complex while retaining the same position of the lower complex band. Because a looped complex is likely to behave differently than a linear complex this indicates that the lower complex band rather represents a linear complex. Based on our observations under the conditions used, we thus conclude BusR, apart from its similarity to Atu1419 in respect to its oligomeric state and the presence of two binding sites, does not induce loop formation *in vitro*.

To our knowledge the quaternary assembly and the mode of regulation is strikingly different from other members of the GntR family and BusR represents a subfamily of its own. BusR shares more overall structural similarity to the MerR family of transcription factors, especially to the c-di-GMP responsive MerR family transcription factor BrlR. Like BusR, BrlR is a dumbbell shaped tetramer with a head-to-tail arrangement and a central coiled-coil motif joining the distal ligand and DNA binding domains. Except for the overall shape, the two transcription factors share little in common. The coiled-coils of BrlR are formed by a dimer of dimers and form a less rigid tetramerization interface compared to BusR. BrlR has two c-di-GMP binding sites structurally unrelated to the c-di-AMP binding site in BusR. While we observe c-di-AMP binding to a regulatory subunit (RCK_C), followed by large conformational changes leading to DNA binding, in BrlR c-di-GMP binding occurs directly at the wHTH domain and the coiled-coil motif, followed by only subtle movement. In this respect, in BrlR binding of c-di-GMP rather resembles an induced or stabilized fit while BusR moves from autoinhibited to activated state upon c-di-AMP binding. Lacking a dsDNA bound structure of BlrR, we refer to BmrR, another transcriptional activator of the MerR family. BmrR dimerizes via a coiled-coil motif. A high degree of conformational plasticity is exhibited by the coiled-coils between apo and DNA bound state (68). Upon binding BmrR strongly bends the DNA in a clamp-like fashion, introducing a significant kink in the DNA that allows for the polymerase to bind subsequently (69). In contrast, the tetrameric coiled-coil of BusR is highly rigid and can be perfectly superimposed from apo to DNA bound state. This rigidity is further transferred to the DNA binding domain upon resealing of helix α_CC_ and α_G_4. We believe that the tetrameric coiled coil mediated assembly is less a means of bending the DNA for transcriptional activation as in case of BmrR and the MerR family, but it adds a second layer of specificity by acting as a molecular ruler between two binding sites separated by a specific distance.

In summary, our biochemical and structural data provide a possible molecular mechanism for the c-di-AMP triggered inactivation of *busA* gene expression by BusR. It explains how the input signal c-di-AMP is translated to gene repression through reconfiguration of the BusR tetramer upon binding of c-di-AMP to the RCK_C dimer. BusR resides in an autoinhibited state by a hydrophobic patch that locks its wHTH domain and prevents high affinity binding to its target in absence of c-di-AMP. This state is shifted to a DNA binding activated state upon binding of its native ligand c-di-AMP. Binding of the second messenger induces movement involving residues of the hydrophobic patch surrounding Trp159 that releases the wHTH domain and allows for strong binding to its operator DNA. This ultimately results in blocking transcription of *busA* and less osmolyte uptake as demonstrated before (37,39). High specificity and selectivity for the downregulated gene is provided by the sequence specificity of the DNA binding domains and the ultimate need for two binding sites in a fixed distance that corresponds to the length of the coiled-coil ruler, achieved by the so far unique domain arrangement of BusR.

## DATA AVAILABILITY

MX and cryoEM structures have been deposited in the RCSB PDB. Accession numbers: 7B5T (BusR fl), 7B5W (RCK_C domain ligand free), 7B5U (RCK_C with c-di-AMP), 7OZ3 (cryoEM structure of BusR-DNA complex (pAB)), 7B5Y (cryoEM structure of the BusR-pAB1 complex). SAXS data have been deposited in the SASBDB with accession numbers SASDK74 (BusR RCK_C domain dimer), SASDK84 (BusR full-length protein), and SASDK94 (BusR:c-di-AMP:pAB1 complex). Other datasets generated and/or analysed during the current study are available from the corresponding author on reasonable request.

## Supplementary Material

gkab736_Supplemental_File
