# Supplementary material for: BusR senses bipartite DNA binding motifs by a unique molecular ruler architecture
Source: Nucleic Acids Res. 2021 Aug 25;49(17):10166–77. doi: 10.1093/nar/gkab736 (PMC8517857; doi:10.1093/nar/gkab736)
Supplement: gkab736_Supplemental_File [file gkab736_Supplemental_File.pdf]

Supplemental Data

BusR Senses Bipartite DNA Binding Motifs by a Unique Molecular Ruler Architecture

Adrian M. Bandera, Joseph Bartho, Katja Lammens, David Jan Drexler, Jasmin Kleinschwärzer, Karl-Peter Hopfner and Gregor Witte

Supplemental Figures

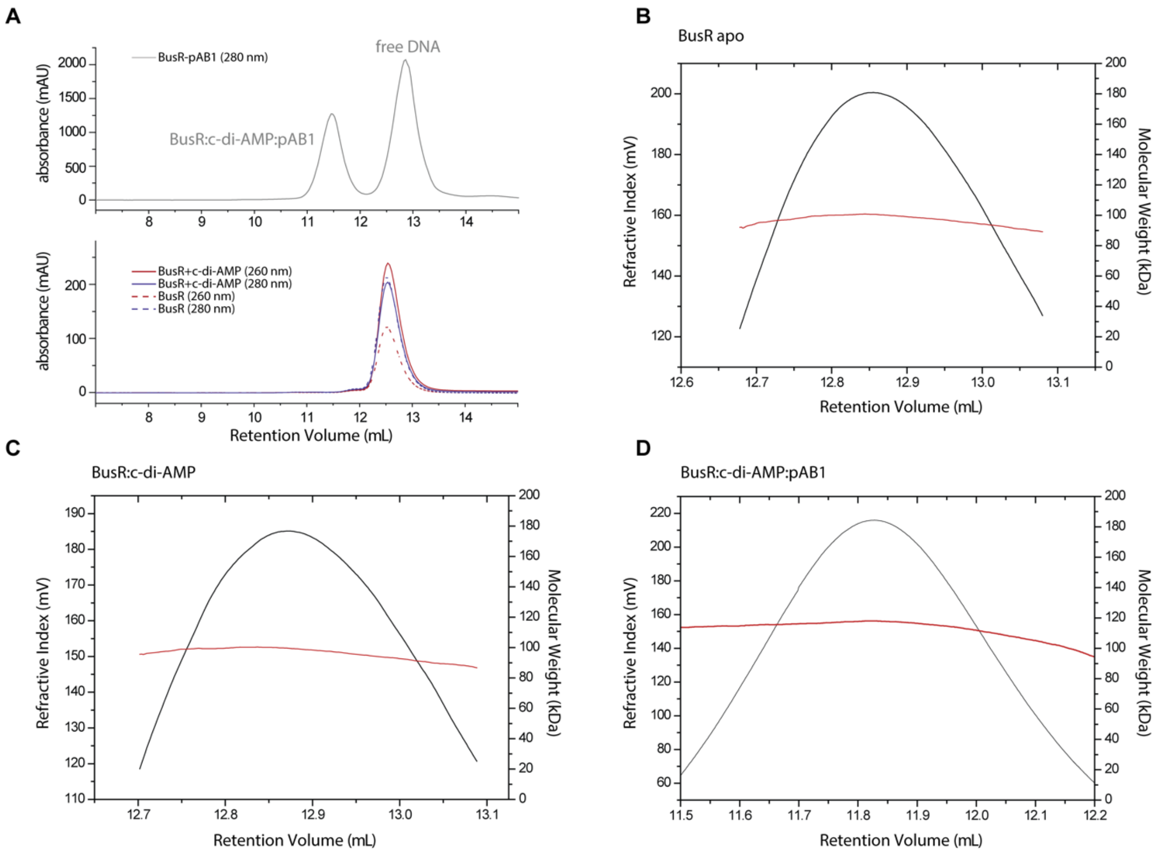

Figure S 1. Determination of Molecular weight and oligomeric state by static light scattering.
Molecular weight determined by size-exclusion chromatography coupled right-angle light scattering using a 24 mL 10/300 Superdex S200 increase column (Cytiva) and an additional refractive index detector (Viscotek).
(A) UV-profile for the BusR:c-di-AMP:pAB1 complex (grey, top), for BusR (dashed line, bottom) at 260 nm (red) and 280 nm (blue) and for BusR with c-di-AMP bound (straight line, bottom). Panels B – D show the refractive Index and the molecular weight for the main peak fractions. (B) BusR has an experimental weight of 97 kDa (Mw/Mn = 1.001, n=1) that corresponds to a tetramer (theoretical Mw = 95.4 kDa). (C) Addition of c-di-AMP does not alter the oligomeric state (measured Mw = 96.3 kDa, theoretical Mw = 96.7 kDa, Mw/Mn = 1.002). (D) A tetramer of BusR binds to a single molecule of double stranded pAB1 – DNA. Experiment conducted in presence of 3-fold excess of DNA (exp. Mw = 116.5 kDa, theoretical Mw (BusR:pAB1) = 123.4 kDa, Mw (BusR:$(pAB1)_2$) = 151.4 kDa, Mw/Mn = 1.000)

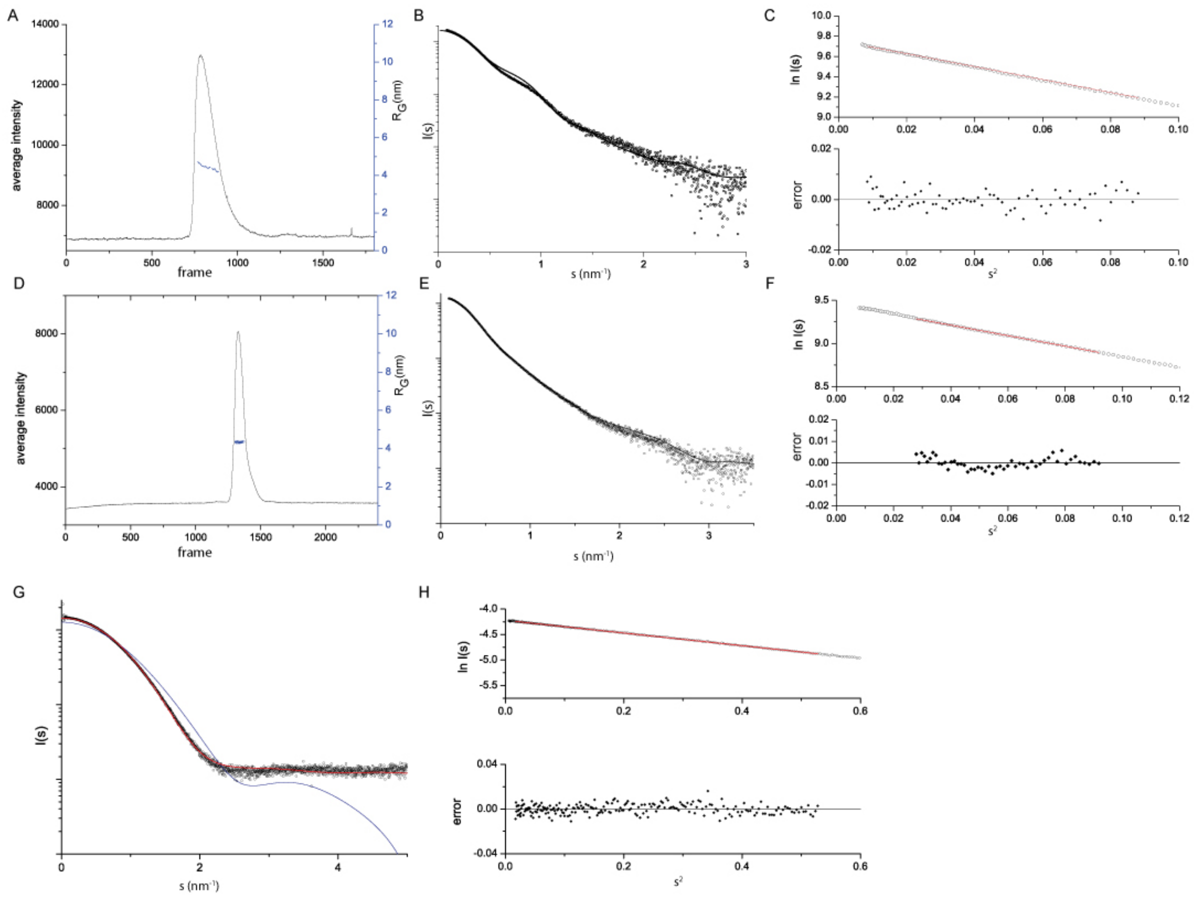

Figure S 2. Small-angle X-ray scattering of BusR.

(A) Chromatogram (average sample scattering vs. retention time = frames) of a size-exclusion coupled SAXS (SEC-SAXS) experiment with 5 mg/mL sample of BusR on a Superdex S200 5/150GL column (20 mM HEPES pH 7.6, 200 mM NaCl, 3% v/v glycerol) with approximated R_G values (blue) of the peak frames.

(B) Averaged sample scattering curve after buffer correction (circles) and the theoretical scattering curve (line) of BusR calculated from our crystal structure using CRYSOL.

(C) Guinier analysis of the scattering data from (B) in the Guinier region $s*R_G<1.3$ (globular particles), indicating that the sample is free of aggregation as can be seen by the homogenous error distribution of the linear regression. (R_G^{BusR} = 4.4 nm)

(D) Chromatogram of SEC-SAXS experiment with a complex of BusR:pAB1:c-di-AMP on a Superdex S200 10/300GL column (20 mM HEPES pH6.5, 100 mM NaCl, 3% glycerol (v/v)) with approximated R_G values (blue) of the peak frames.

(E) Averaged sample scattering curve after buffer correction (circles) and the theoretical scattering curve (line) of the BusR:pAB1:c-di-AMP complex calculated from our cryo-EM structure (χ^2 = 5.8)

(F) shows the Guinier analysis of the scattering data from (E) in the Guinier region with errors ($R_G^{complex}$ = 4.28 nm)

(G) Scattering curve of the isolated RCK_C domain in solution (batch measurement, buffer 100 mM NaCl, 30 mM HEPES, pH 7.5) with calculated scattering curves of a RCK_C monomer (blue) and RCK_C dimer (red, χ^2 = 5.8). The scattering curve is only compatible with the presence of RCK_C dimers in solution.

(H) Guinier analysis of the scattering data from (G) with errors ($R_G^{RCK\_C\_dimer}$ = 1.9 nm).

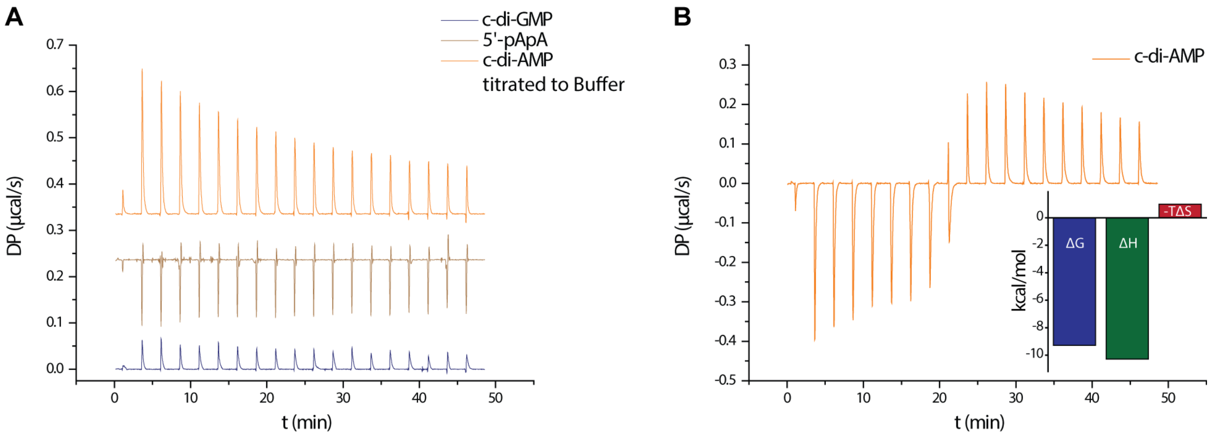

Figure S 3. Raw data for binding analysis of c-di-AMP by ITC, related to Figure 1(C).

(A) ITC measurement raw data of 20 µM full length BusR titrated with 400 µM c-di-GMP (blue) or 5'-pApA (brown). A control of c-di-AMP titrated to buffer results in a strong signal and is therefore displayed as well (orange) and is used as control reference for correction of (B) – see Fig. 1C.

(B) ITC measurement raw data (uncorrected) of 400 µM c-di-AMP titrated to 20 µM full length BusR and respective corresponding signature plot of the corrected data – Fig. 1C.

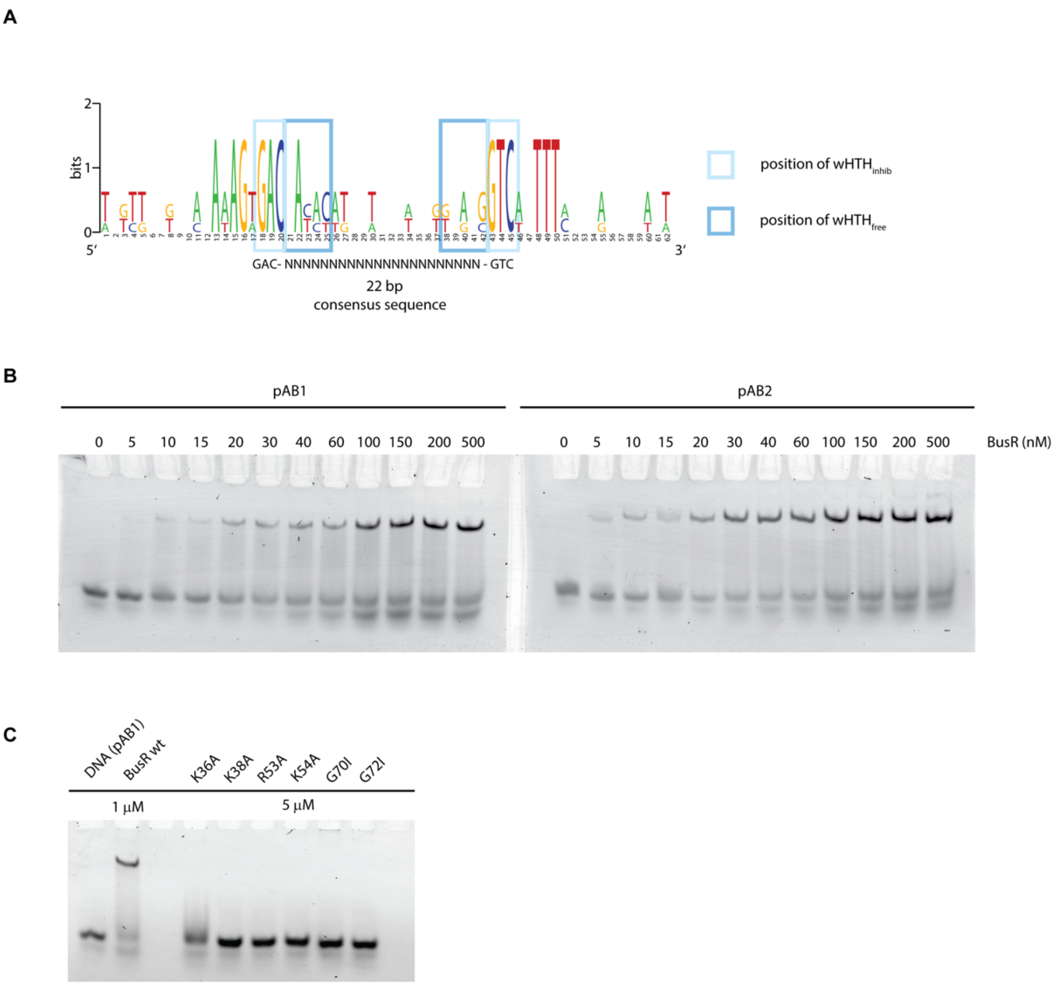

Figure S 4. Additional experimental data on BusR DNA binding by EMSA.

(A) Logo plot of aligned binding sites of four experimentally confirmed operator sequences of BusR from *Lactococcus lactis, Tetragenococcus halophilus*, and two sequences from *Streptococcus agalactiae* (created with the Berkeley WebLogo server[1]).

(B) BusR titrated to pAB1-DNA (left) and pAB2-DNA (right) in presence of c-di-AMP (20 nM DNA, 10 µM c-di-AMP, n = 3). Both binding sites are bound with comparable affinity.

(C) BusR mutants with point mutations in the wHTH motif in comparison to BusR wt by EMSA. The depicted mutants all show severe binding deficiency compared to the wildtype (20 nM DNA, 10 µM c-di-AMP, n = 3).

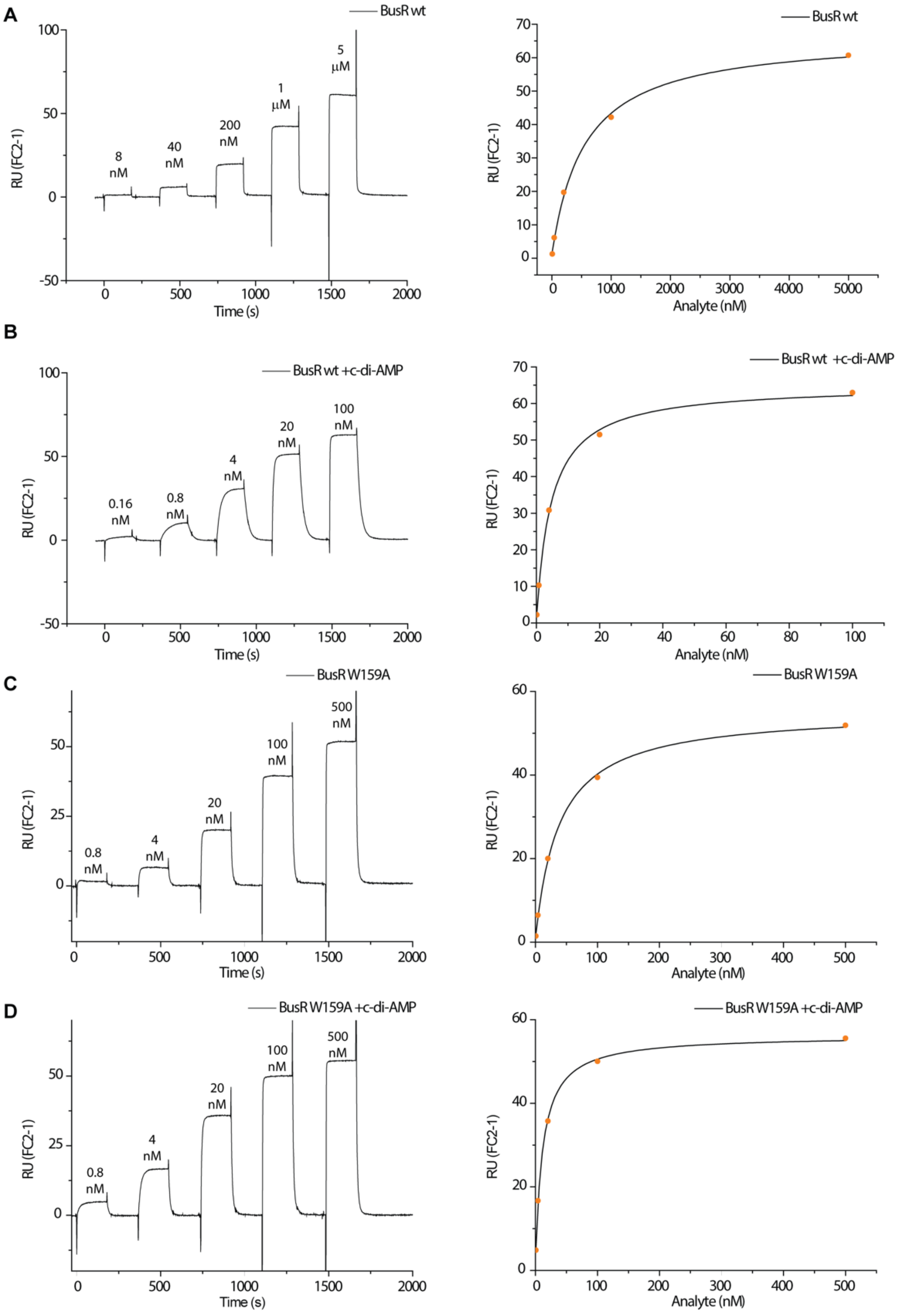

Figure S 5. Raw data of BusR DNA binding by SPR.
Representative SPR raw data (left) of BusR titrated to pAB1 (on chip) in single-cycle assays and their corresponding steady state affinity fit for a 1:1 binding model (right). For all experiments we performed independent triplicate measurements.
(A) BusR wt (0.8 nM – 5 µM analyte concentration) in absence of c-di-AMP, K_D = 577 ± 55 nM.
(B) BusR wt (0.16 nM – 100 nM) in presence of 10 µM c-di-AMP (10 µM), K_D = 5.1 ± 0.4 nM.
(C) BusR W159A (0.8 nM – 500 nM) in absence of c-di-AMP, K_D = 11 ± 2 nM.
(D) BusR W159A (0.8 nM – 500 nM) in presence of c-di-AMP (10 µM), K_D = 34 ± 8 nM.

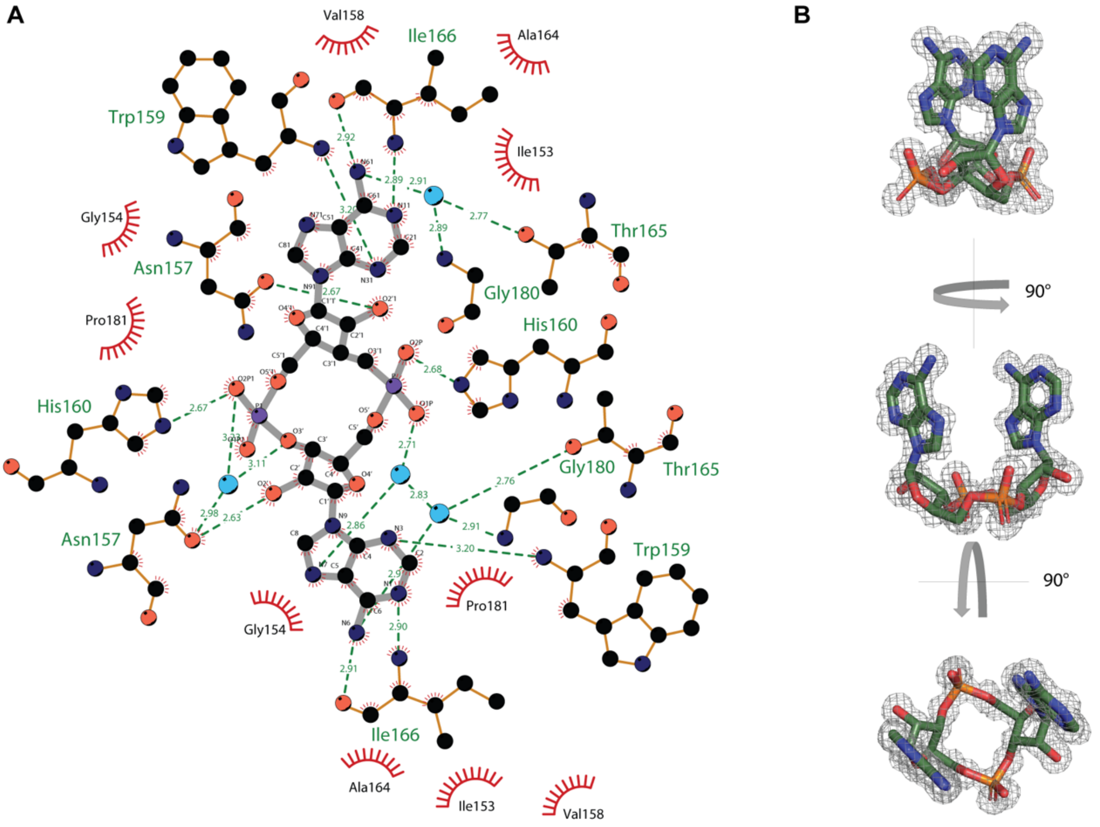

Figure S 6. Ligand coordination plot and polder map, related to figure 2.
(A) Schematic plot of the c-di-AMP coordination by the RCK_C domain, calculated with ligplot [2].
(B) Polder map of c-di-AMP in the binding pocket at 3σ contour level from different angles [3].

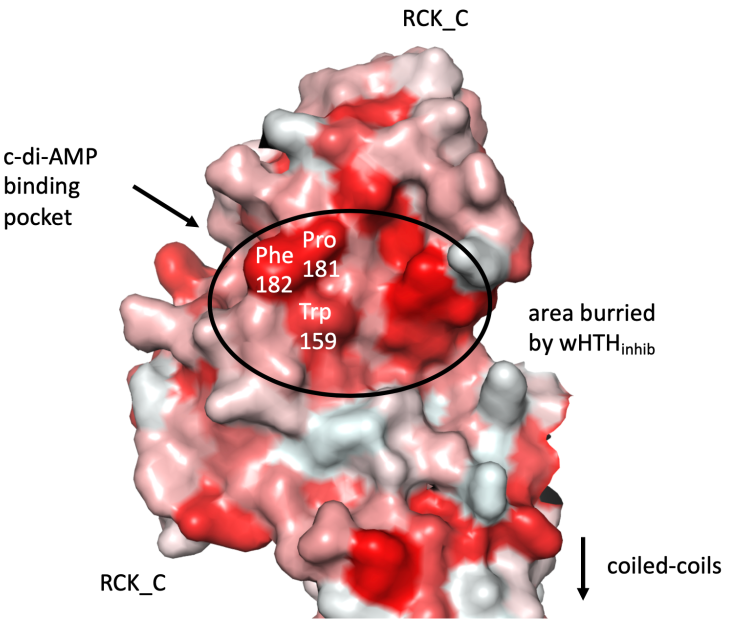

Figure S 7. Hydrophobicity of the RCK_C – wHTH$_{inhib}$ interface.
Side view of the RCK_C domain of full length BusR as surface representation colored according to hydrophobicity (red = hydrophobic). Both DNA binding domains have been omitted for clarity reasons. The circle indicates the area that is buried in the interface with wHTH$_{inhib}$.

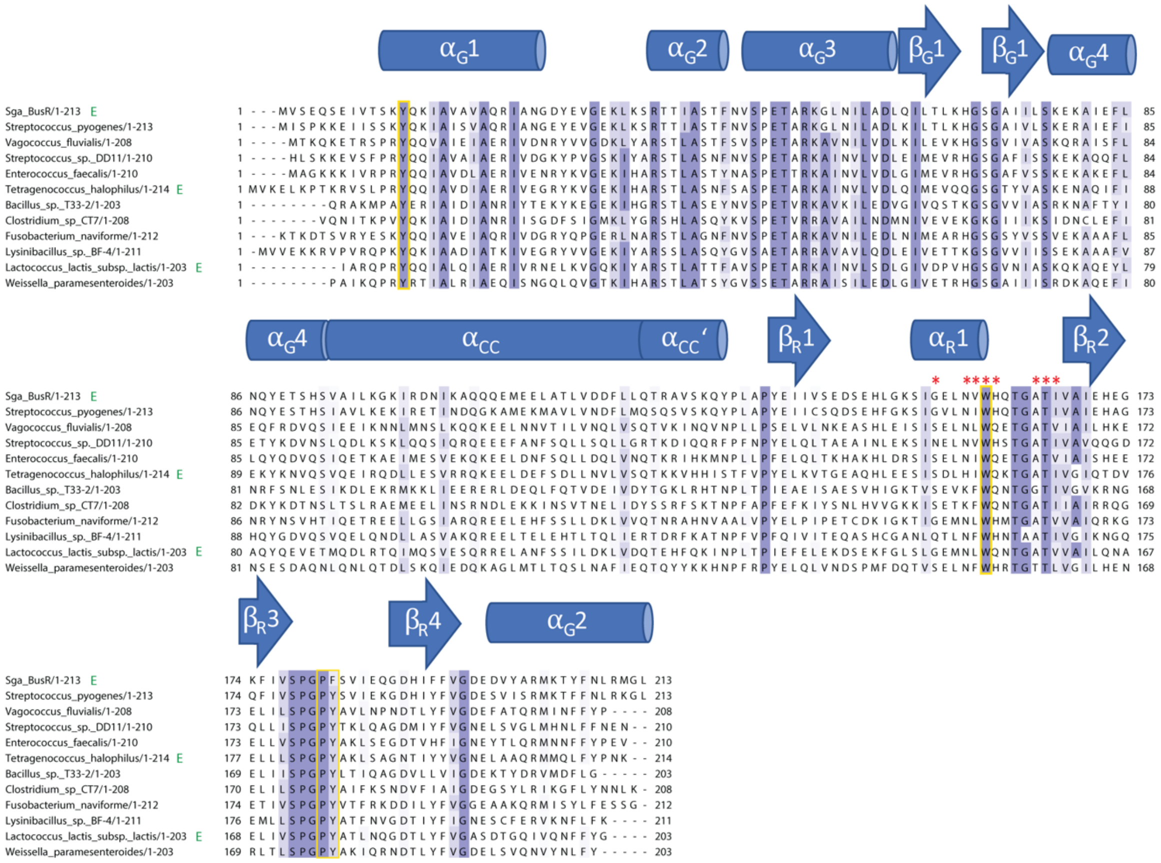

Figure S 8. Multiple Sequence Alignment of BusR homologues.
Putative BusR sequences from different species. The resulting sequences include also experimentally described homologues (green E). The resulting multiple sequence alignment is colored according to conservation. Residues involved in the RCK_C - $wHTH_{inhib}$ signaling interface are highlighted with a yellow box. Residues involved in c-di-AMP coordination are marked by a red asterisk.

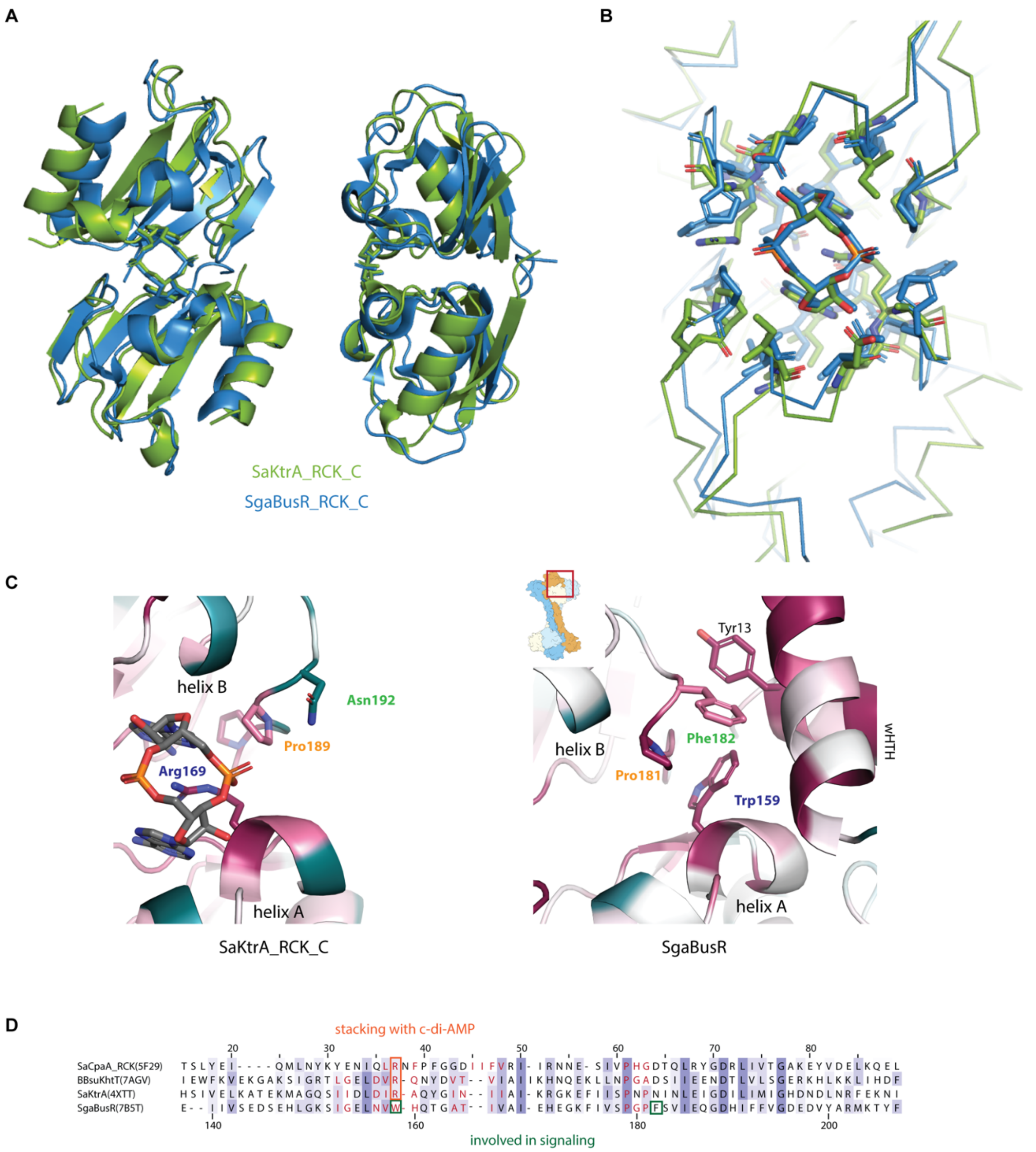

Figure S 9. Comparison of RCK_C domains from *S. agalactiae* BusR and *S. aureus* KtrA.

(A) Superposition of the RCK_C domains from the different proteins BusR and KtrA shows a high structural similarity (RMSD = 1.379 Å).

(B) Stick representation of the c-di-AMP binding sites. The ligand binding sites share very similar main chain geometry while most sidechains are not conserved

(C) Close-up on "signaling residues" in SaKtrA (left) and their counterpart in apo BusR (right). Residues are colored according to conservation generated with the ConSurf server from 150 sequences. Highly conserved residues are colored in dark purple, high variability is presented in cyan. While SaKtrA Arg169 stacks with c-di-AMP, the sidechain of BusR Trp159 is not involved in c-di-AMP coordination but coordinates a conserved hydrophobic patch including Tyr13, Phe182 and Pro181. SaKtrA Phe192, the equivalent of BusR Phe182 is not conserved, as no regulatory hydrophobic patch exists.

(D) Sequence alignment of c-di-AMP binding and similar structurally characterized RCK_C domains.

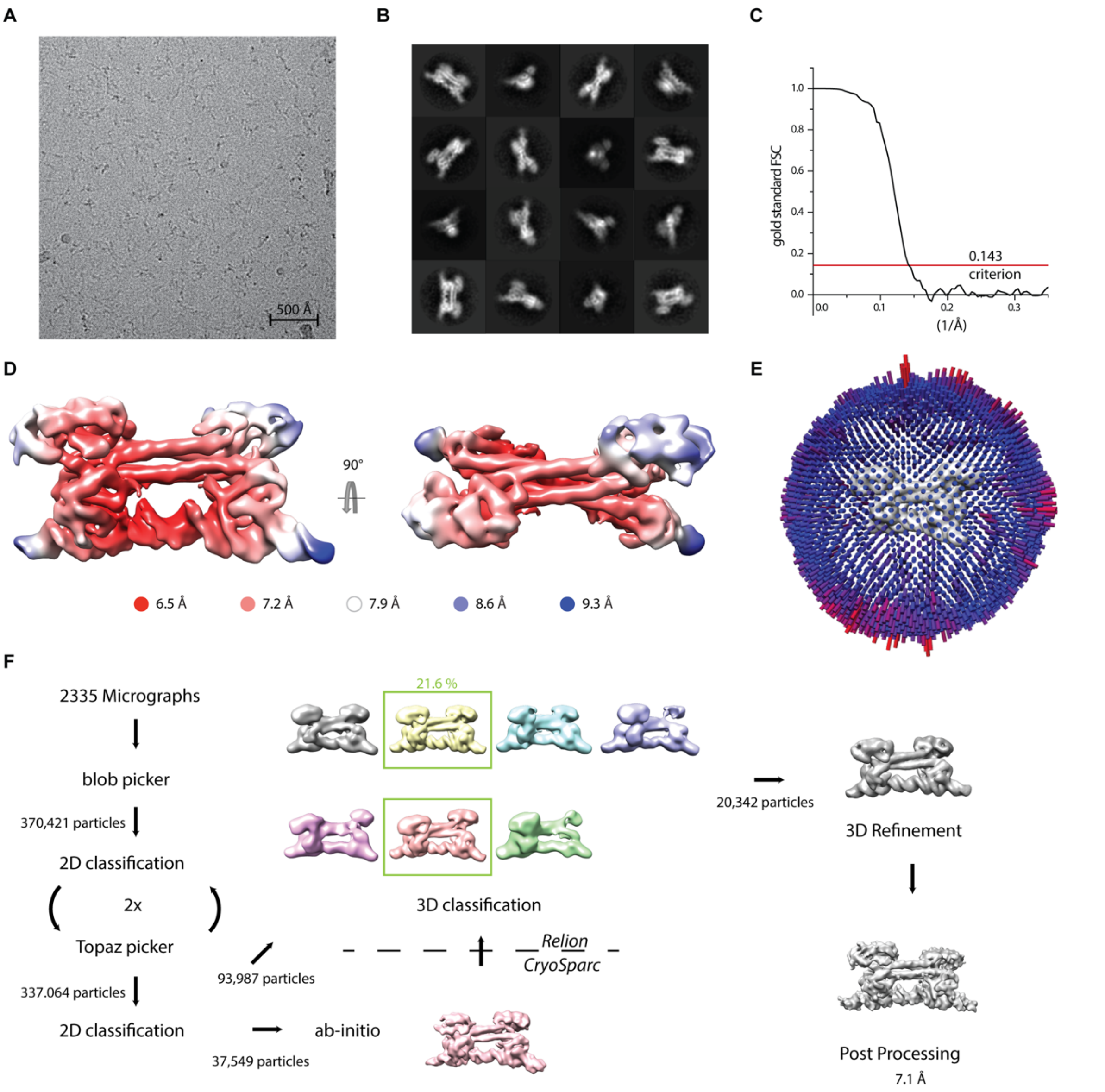

Figure S 10. CryoEM processing of the BusR:c-di-AMP:pAB1 complex.
(A) Representative micrograph at 30° tilt angle.
(B) 16 distinct and highly populated 2D classes from the final reconstruction.
(C) Gold-standard Fourier shell correlation curve. The red line represents the 0.143 criterion which proposes 7.1 Å average resolution.
(D) Variability in local resolution ranging from 6.5 Å (dark red) to 9.3 Å (dark blue). Calculated using Relion 3.1
(E) Overall angular distribution of the particles used for final reconstruction.
(F) Schematic Processing scheme leads to the final reconstruction with 20,342 particles and C1 symmetry.

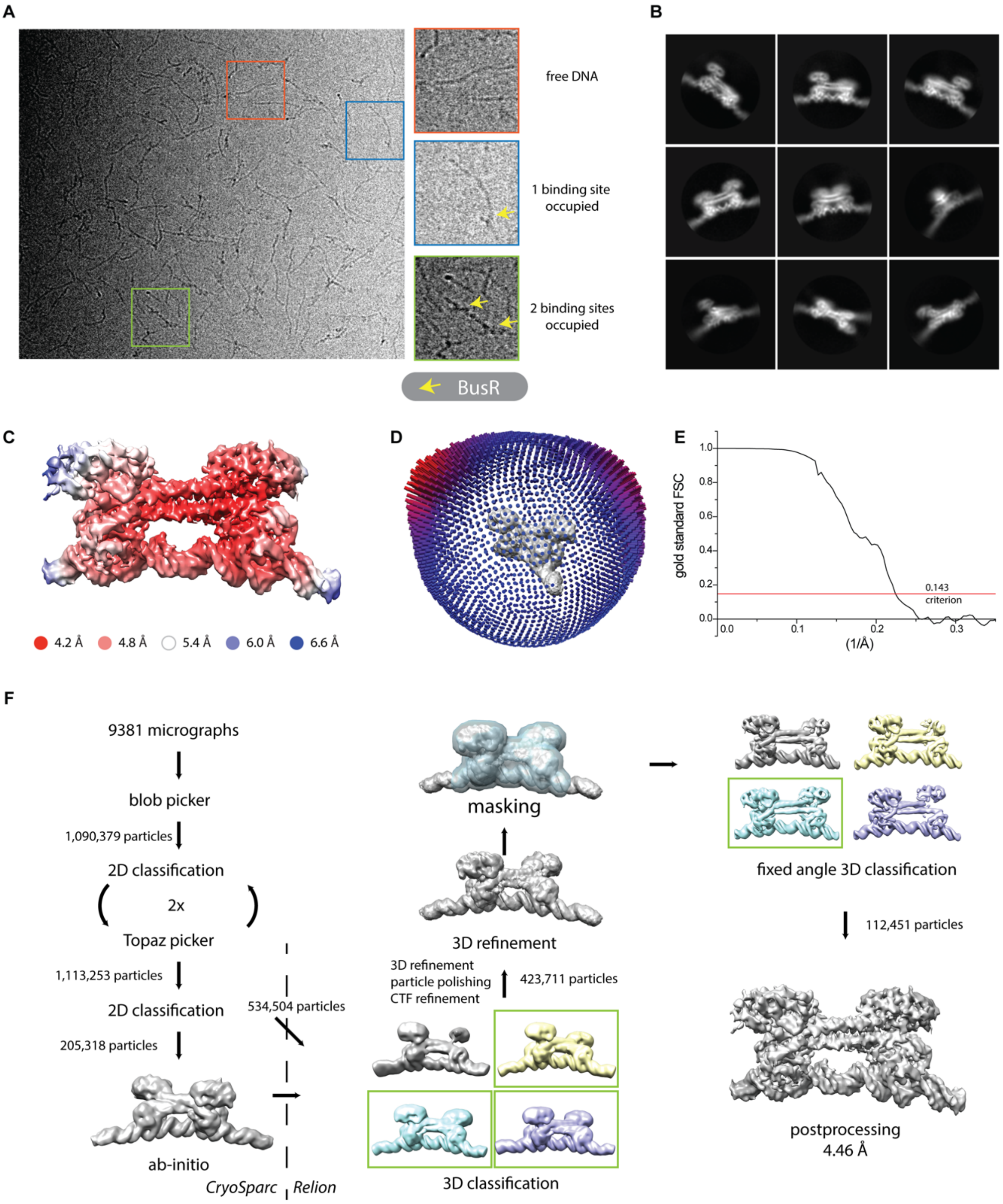

Figure S 11. CryoEM processing of the BusR:c-di-AMP:pbusA complex and additional data related to figure 3
(A) representative micrograph and highlighted observed species.
(B) Representative 2D classes from the final reconstruction.
(C) Variability in local resolution. Resoltion ranging from 4.2 Å (dark red) to 6.6 Å (dark blue) according to Relion 3.1.2.
(D) Overall angular distribution of the particles used for final reconstruction.
(E) Gold-standard Fourier shell correlation curve. The red line represents the 0.143 criterion which proposes 4.46 Å average resolution (calculated using Relion 3.1)
(F) Complete processing scheme for BusR:c-di-AMP:pAB.

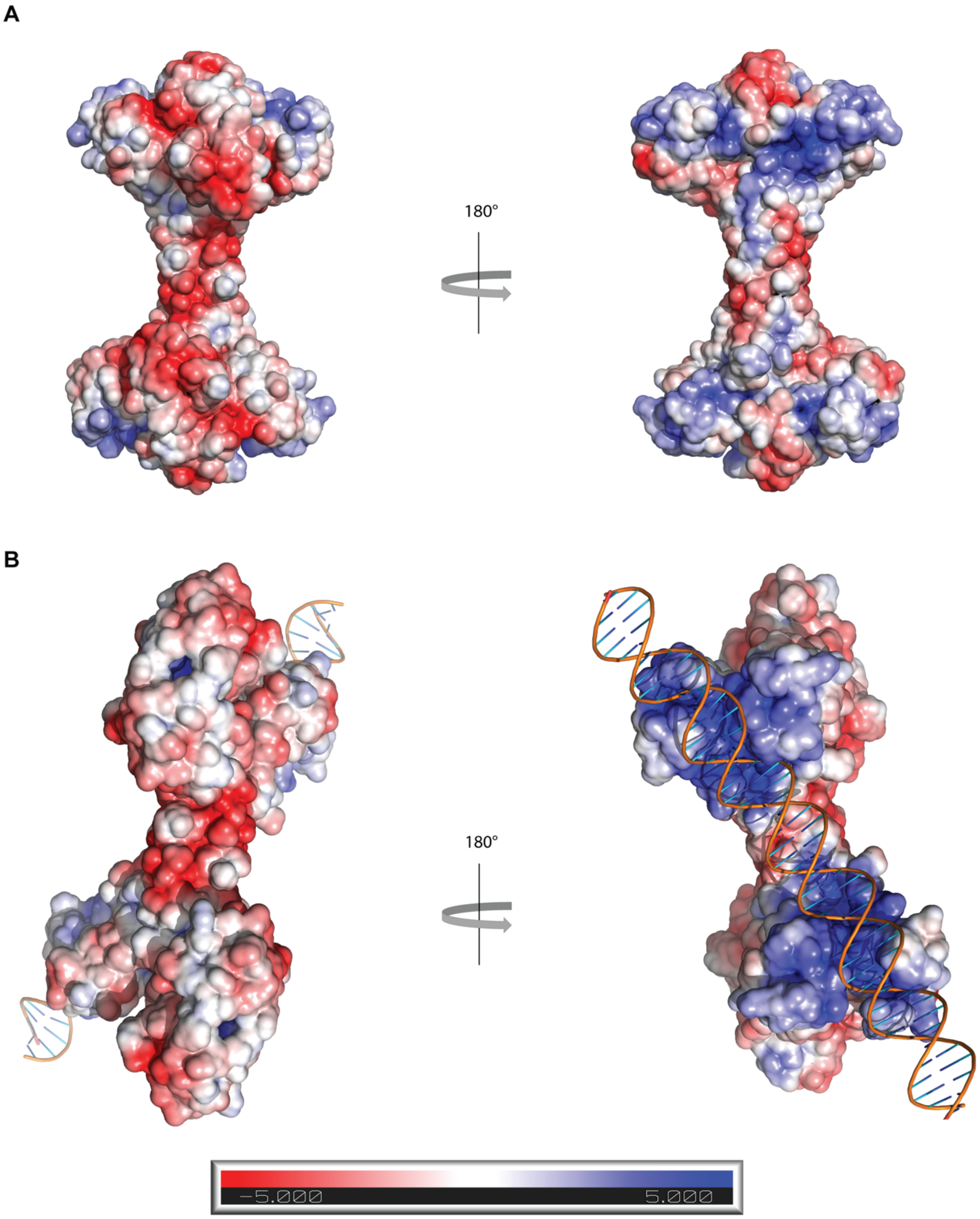

Figure S 12. Surface accessible electrostatic surface of BusR.
All solvent accessible electrostatic surfaces (ranging from -5 KT/e to 5 KT/e) were calculated using the Adaptive Poisson-Boltzmann Solver (APBS) plug-in in pymol [4].
(A) Apo BusR reveals an asymmetric distribution of electrostatic potential.
(B) When bound to DNA the negative charge of the DNA's phosphate backbone is directed towards the positively charged surface of BusR surrounding the DNA. The DNA was removed from the model during APBS calculation.

Table S1. Crystallographic Data and Refinement Statistics

Protein	BusR_fl_wt	RCK_C-cdA	His_6-RCK_C
**Data collection**			
**Beamline**	EMBL-P13	EMBL-P14	EMBL-P13
**Wavelength (Å)**	0.9797	0.9763	0.9
**Resolution range**	48.22 - 2.802 (2.902 - 2.802)	17.48 - 1.2 (1.23 - 1.2)	18.0 - 1.001 (1.03 - 1.00)
**Space group**	I 4	P 1 21 1	C 1 2 1
**Cell dimensions**			
**a, *b*, *c* (Å)**	114.18 114.18240.48	33.16 60.95 35.71	46.26 40.41 41.01
α, β, γ (°)	90 90 90	90 101.73 90	90 103.21 90
**Total reflections**	518066 (36988)	285772 (16873)	251457 (15270)
**Unique reflections**	74011 (5409)	41498 (2859)	39372 (2881)
**Multiplicity**	6.9 (6.83)	6.9 (5.9)	6.4 (5.3)
**Completeness (%)**	99.1 (97.7)	95.4 (88.3)	99.1 (97.0)
**I/sigma(I)**	19.4 (1.02)	36.6 (14.9)	21.6 (1.9)
**Wilson B-factor**	101.5	12.6	14.4
**R-meas**	8.0 (164.6)	3.4 (11)	3.2 (96.6)
**CC1/2**	100.0 (61.5)	100 (99.3)	100 (77.3)
**Refinement**			
**Resolution**	2.8	1.2	1.0
**Reflections used in refinement**	37333 (3694)	41497	39369
**Reflections used for R-free**	1827 (154)	1923	1906
**R-work**	25.7 (41.2)	13.1 (11.4)	19.9 (34.3)
**R-free**	26.9 (42.9)	15.5 (13.4)	22.6 (34.6)
**Molecules (asu)**	1 tetramer	1 dimer	1 monomer
**Number of non-hydrogen atoms**	6528	1585	898
**macromolecules**	6506	1307	794
**solvent**	22	278	104
**Protein residues**	828	157	97
**RMS(bonds)**	0.005	0.006	0.009
**RMS(angles)**	0.96	1.28	1.28
**Ramachandran favored (%)**	95.1	98.69	96.84
**Ramachandran allowed (%)**	4.8	1.31	2.11
**Ramachandran outliers (%)**	0.1	0.00	1.05
**Rotamer outliers (%)**	3.5	0.00	0.00
**Clashscore**	11.5	1.96	0.00
**Average B-factor**	127.9	12.90	20.38
**macromolecules**	128.0	10.26	19.08
**solvent**	98.1	25.33	30.28
**Number of TLS groups**	12		
**PDB ID**	7B5T	7B5U	7B5W

rmsd, root-mean-square deviation. asu, asymmetric unit. Values in parentheses are for the last resolution shell

Table S2. EM Data Collection, 3D Reconstruction, and Model Refinement Statistics

	S. agalactiae **BusR:c-di-AMP:pAB complex**	***S. agalactiae*** **BusR:c-di-AMP: pAB1**
**Data collection and processing**		
**Magnification**	130000	130000
**Voltage (kV)**	300	300
**Electron exposure (e^{-}/Å^2)**	45	45
**Defocus range (μm)**	-1.1 to -2.6	-1.0 to -2.8
**Pixel size (Å)**	1.046	1.059
**Symmetry imposed**	C1	C1
**Initial particle images (no.)**	1,090,379	370,421
**Final particle images (no.)**	112,451	20,342
**Map resolution (Å) / FSC threshold**	4.46 / 0.143	7.1 / 0.143
**Map-resolution range (Å)**	4.2 - 6.6	6.5 - 9.3
**Refinement**		
**Initial model used (PDB code)**	This work	This work
**Model resolution (Å)**	4.0	7.1
**FSC threshold**	0 / 0.143 / 0.5	0 / 0.143 / 0.5
**Model-resolution range (Å)**	3.8 / 3.9 / 4.5	5.5 / 6.3 / 7.7
**Map-sharpening *B* factor (Å^2)**	- 177.2	-492.7
**Model composition**		
**Nonhydrogen**	8209	8434
**Protein residues**	804	818
**Nucleotides**	88	92
**Ligands**	c-di-AMP (2x)	c-di-AMP (2x)
**Mean *B* factors (Å^2)**		
**Protein**	127.7	226.7
**Ligand**	141.9	319.5
**Nucleotide**	158.5	256.3
**R.m.s. deviations**		
**Bond lengths (Å)**	0.009	0.009
**Bond angles (°)**	1.35	1.6
**Validation**		
**MolProbity score**	1.75	2.46
**Clashscore**	8.38	13.56
**Poor rotamers (%)**	0.29	3.26
**Ramachandran plot**		
**Favored (%)**	95.7	93.46
**Allowed (%)**	4.3	5.93
**Disallowed (%)**	0	0.62
**PDB ID**	7OZ3	7B5Y

Table S 3. Oligos (5' – 3')

purpose	name	sequence	modification
cloning	Sga BusR f	TCTTGAAGTCCTCTTTCAGGGACCCATGGTTTCTGAACAATCTGAAATTGTAACATC	
	Sga BusR r	TGGCACCAGAGCGAGCTCAAGCCCCATCCTTAAGTTAAAATAGGTTTTCA	
	Sga RCK_C f	CTTGAAGTCCTCTTTCAGGGACCCCTAGCACCATATGAAATTATTG	
	QC W159A f	TAGGAGAATTAAATGTAGCGCATCAAACTGGTGCGAC	
	QC W159A r	GTCGCACCAGTTTGATGCGCTACATTTAATTCTCCTA	
	QC_K36A f	GTTGGAGAGAAGTTGGCATCTAGAACAACTATT	
	QC_K36A r	AATAGTTGTTCTAGATGCCAACTTCTCTCCAAC	
	QC_R38A f	GAGAAGTTGAAATCTGCAACAACTATTGCTTCA	
	QC_R38A r	TGAAGCAATAGTTGTTGCAGATTTCAACTTCTC	
	QC_R53A f	TCACCAGAAACAGCAGCTAAGGGTCTTAATATT	
	QC_R53A r	AATATTAAGACCCTTAGCTGCTGTTTCTGGTGA	
	QC_K54A f	CCAGAAACAGCACGTGCGGGTCTTAATATTTTA	
	QC_K54A r	CTAAAATATTAAGACCCGCACGTGCTGTTTCTGG	
	QC_G70I f	TTAACTTTAAAGCATATCAGTGGAGCCATTATT	
	QC_G70I r	AATAATGGCTCCACTGATATGCTTTAAAGTTAA	
	QC_G72I f	TTAAAGCATGGCAGTATAGCCATTATTCTTTCT	
	QC_G72I r	AGAAAGAATAATGGCTATACTGCCATGCTTTAA	
	QC_pAB1_BS1-f	GAGTAAAAAAGTATGTACCCTTTTACGATAAC	
	QC_pAB1_BS1-r	GTTATCGTAAAAGGGTACATACTTTTTTACTC	
	QC_pAB1_BS2-f	GTGTCTTATCTATAAAGTATGGTTGAGTTATCG	
	QC_pAB1_BS2-r	CGATAACTCAACCATACTTTATAGATAAGACAC	
Cloning & Amplification For cryoEM	pAB_f	ATAAAGCTTCTCTAAGCAAGGTG	
	pAB_r	ATAGGATCCGTGTCTTATCTATAAAGTG	
EMSA	pAB1_for	ATAAAGTGACGTTAAAGTATCGTAAAAGGGTAGTCACTTTTTTACT	5'-FAM
	pAB1_rev	AGTAAAAAAGTGACTACCCTTTTACGATACTTTAACGTCACTTTAT	
	pAB1_f 0BS_for	ATAAAGTATGGTTAAAGTATCGTAAAAGGGTACATACTTTTTTACT	5'-FAM
	pAB1_r 0BS_rev	AGTAAAAAAGTATGTACCCTTTTACGATACTTTAACCATACTTTAT	
	pAB1_1BS-A_for	ATAAAGTATGGTTAAAGTATCGTAAAAGGGTAGTCACTTTTTTACT	5'-FAM
	pAB1_1BS-A_rev	AGTAAAAAAGTGACTACCCTTTTACGATACTTTAACCATACTTTAT	
	pAB1_1BS-B_for	ATAAAGTGACGTTAAAGTATCGTAAAAGGGTACATACTTTTTTACT	5'-FAM
	pAB1_1BS-B_rev	AGTAAAAAAGTATGTACCCTTTTACGATACTTTAACGTCACTTTAT	
	pAB1_f_5bp_for	ATAAAGTGACGTTAAAGTATCGTCGATTAAAAGGGTAGTCACTTTTTTACT	5'-FAM
	pAB1_r_5bp_rev	AGTAAAAAAGTGACTACCCTTTTAATCGACGATACTTTAACGTCACTTTAT	
	pAB1_f_-10bp_for	ATAAAGTGACGTTAAAAGGGTAGTCACTTTTTTACT	5'-FAM
	pAB1_r_-10bp_rev	AGTAAAAAAGTGACTACCCTTTTAACGTCACTTTAT	
	ref_f	GGTCATACTTCCTAAGTCACCGGTATGGTAAGCAGGTAGACCTTCGA	5'-FAM
	ref_r	TCGAAGGTCTACCTGCTTACCATACCGGTGACTTAGGAAGTATGACC	
	pbusA_FAM_f	AAGCTTCTCTAAGCAAGGTG	5'FAM
	pbusA_FAM_r	GGATCCGTGTCTTATCTATAAAGTG	5'FAM
cryoEM & SAXS	pAB1_EM_f	CGGTAAAGTGACGTTAAAGTATCGTAAAAGGGTAGTCACTTTTCGG	
	pAB1_EM_r	CCGAAAAGTGACTACCCTTTTACGATACTTTAACGTCACTTTACCG	

Supplemental Methods

A second cryoEM structure of the BusR:DNA complex was solved using shorter DNA with only a single binding site. For grid preparation full-length BusR was incubated with excess c-di-AMP and pAB1 DNA and purified via size exclusion chromatography (Superdex S200 10/300) equilibrated in 20 mM HEPES pH 6.5, 100 mM NaCl. Peak fractions, corresponding to approx. 0.5 mg/mL were pooled. Prior to grid preparation β-octyl glucoside was added to a final concentration of 0.05%. 4.5 µL of sample was applied to plasma cleaned (GloCube, Quorum) UltrAuFoil R2/2 200 mesh grids (Quantifoil), then plunge frozen in liquid ethane using a Leica EM GP. Data was acquired using a Titan Krios transmission electron microscope (Thermo Fisher Scientific) operated at 300 keV, with a Gatan K2 Summit detector operated in counting mode, and Gatan GIF Quantum energy filter. EPU software (TFS) was used for automated acquisition. 3 Datasets at different tilt angles of 20°, 25° and 30° were collected with a total of 2,335 micrographs, with a nominal magnification of 130,000x, calibrated pixel size of 1.059 Å, defocus range of -1.0 to -2.8 µm, and a total dose of 46 $e^-/Å^2$ over 40 frames. For data processing all micrographs were aligned using Motioncor2 [5]. The subsequent steps were done in cryoSPARC v2.15 [6]. Local CTF estimation was done with Patch CTF implemented in cryoSPARC. Initial particles were picked using blob picker. These particles were 2D classified, and high quality and diverse classes were selected and low pass filtered to 20 Å for training of the topaz neural network picker embedded in cryoSPARC [7,8]. The topaz picked particles were extracted and resubjected to 2D classification and topaz training. Final extraction was done with a box size of 220 pixels and particles were subjected to 2D classification. Classes of good resolution were selected and used to generate three initial 3D models (C1 symmetry). The best volume was used as a reference in Relion 3.1 for further 3D classification [9]. The best 3D classes were selected and used for 3D refinement followed by post processing.

Model building was done by using the crystallographic full-length structure of BusR (this work) and dissecting every monomer into the wHTH domain and the RCK_C domain. The coiled-coil domain

with parts of all four monomers was taken as a single entity. These models were rigid body fitted into the cryo-EM map using UCSF Chimera. Coot was used for further building. The linker between the coiled-coils and the RCK_C domain was freely built into the density. An ideal B-DNA based on the sequence of pAB1 was generated and bent to fit the map.

Supplemental References

1 Crooks, G. E., Hon, G., Chandonia, J. M. & Brenner, S. E. WebLogo: a sequence logo generator. *Genome Res* **14**, 1188-1190, doi:10.1101/gr.849004 (2004).

2 Wallace, A. C., Laskowski, R. A. & Thornton, J. M. LIGPLOT: a program to generate schematic diagrams of protein-ligand interactions. *Protein Eng* **8**, 127-134, doi:10.1093/protein/8.2.127 (1995).

3 Liebschner, D. *et al.* Polder maps: improving OMIT maps by excluding bulk solvent. *Acta Crystallogr D Struct Biol* **73**, 148-157, doi:10.1107/S2059798316018210 (2017).

4 Baker, N. A., Sept, D., Joseph, S., Holst, M. J. & McCammon, J. A. Electrostatics of nanosystems: application to microtubules and the ribosome. *Proc Natl Acad Sci U S A* **98**, 10037-10041, doi:10.1073/pnas.181342398 (2001).

5 Zheng, S. Q. *et al.* MotionCor2: anisotropic correction of beam-induced motion for improved cryo-electron microscopy. *Nat Methods* **14**, 331-332, doi:10.1038/nmeth.4193 (2017).

6 Punjani, A., Rubinstein, J. L., Fleet, D. J. & Brubaker, M. A. cryoSPARC: algorithms for rapid unsupervised cryo-EM structure determination. *Nat Methods* **14**, 290-296, doi:10.1038/nmeth.4169 (2017).

7 Bepler, T. *et al.* Positive-unlabeled convolutional neural networks for particle picking in cryo-electron micrographs. *Nat Methods* **16**, 1153-1160, doi:10.1038/s41592-019-0575-8 (2019).

8 Bepler, T., Kelley, K., Noble, A. J. & Berger, B. Topaz-Denoise: general deep denoising models for cryoEM and cryoET. *Nat Commun* **11**, 5208, doi:10.1038/s41467-020-18952-1 (2020).

9 Scheres, S. H. RELION: implementation of a Bayesian approach to cryo-EM structure determination. *J Struct Biol* **180**, 519-530, doi:10.1016/j.jsb.2012.09.006 (2012).
